# Integrated GIS-AHP based assessment of earthquake vulnerability and risk for urban residential buildings in Muscat, Sultanate of Oman

**DOI:** 10.1038/s41598-025-17618-6

**Published:** 2025-08-30

**Authors:** Abdullah Ansari, Issa El-Hussain, Ahmed Deif, Adel M. E. Mohamed, Yousuf Al-Shijbi, Khalifa Al-Jabri, Pranjal Mandhaniya, Jong-Han Lee, Ayed E. Alluqmani, E. Mutaz, Hajar Al-Qayoudhi, Wiam Al-Abdulsalam, Al-Anoud Al-Kharusi, Majid Al-Musalhi, Shima Al-Balushi

**Affiliations:** 1 Earthquake Monitoring Center, Sultan Qaboos University, PC: 123 Al Khoudh, Muscat, Oman; 2 Department of Civil and Architectural Engineering, Sultan Qaboos University, Muscat, PC: 123 Al Khoudh, Muscat, Oman; 3 Department of Mechanical and Industrial Engineering, Norwegian University of Science and Technology Trondheim, Trondheim, Norway; 4 Department of Civil Engineering, Inha University, Incheon, 22212 Republic of Korea; 5 Department of Smart City Engineering, Inha University, Incheon, 22212 Republic of Korea; 6 Department of Civil Engineering, Faculty of Engineering, Islamic University of Madinah, Al-Madinah Al-Munawarah, Saudi Arabia; 7 School of Civil Engineering, The University of Queensland, Brisbane, Australia

**Keywords:** Seismic vulnerability, Risk, Residential buildings, Multi-criteria decision making, GIS-AHP, Damage assessment, Environmental social sciences, Natural hazards

## Abstract

This study presents a cutting-edge framework for assessing earthquake vulnerability and risk in residential areas of Al-Seeb, Muscat Governorate (Sultanate of Oman). Drawing upon a rich dataset encompassing seismic, geotechnical, structural, environmental, and socioeconomic parameters, thematic vulnerability maps were developed using a GIS-based analytic hierarchy process (GIS-AHP). These were systematically integrated to produce comprehensive risk matrices. It has been noted that the Rusayl industrial estate emerged as a critical hotspot due to the clustering of industrial facilities and fuel stations near densely populated zones, amplifying hazard proximity. Physical vulnerability patterns largely mirrored geotechnical characteristics but were further shaped by urban density and infrastructure quality. A risk index was derived for each grid cell, enabling the formulation of a detailed microzonation map that categorizes Al-Seeb into five distinct risk zones. Central urban areas like Al-Hail North and segments of Old Al-Khoud plunged within the highest vulnerability category, while parts of Al-Mawaleh North exhibited a blend of high and very high risk, primarily due to aging infrastructure and insufficient retrofitting. Interestingly, Al-Maabilah showed signs of structural resilience, with 60% of buildings experiencing minor damage and only 20% sustaining major impacts. The robust database generated offers critical insights for policymakers, planners, and stakeholders, serving as a strategic tool for informed decision-making. The study’s outcomes align well with Oman Vision 2040 and contribute directly to the goals of sustainable and disaster-resilient urban development outlined in the United Nation’s Sustainable Development Goal (UN-SDG) 11. By embedding these insights into national urban planning and construction policies, the study supports risk-informed development and financial risk reduction for any futuristic projects in the Sultanate of Oman. In response to the shortcomings and delays of previous research, the present study is the first of its kind in the Arabian region, significantly contributing to the establishment of a sustainable and resilient Omani society.

## Introduction

Annually, significant earthquakes worldwide result in severe damage to urban residential buildings due to inadequate planning and engineering, and poor-quality construction practices. This leads to loss of life, property, and economic resources on a national scale^1–6^. With the ongoing trends of globalization, urbanization, and industrial expansion, the migration of people to urban environment and the development of industrial zones exerts substantial pressure on existing residential infrastructure^7^. Consequently, the performance of urban environments following disasters becomes increasingly challenging. Addressing this issue requires defined solutions that promote sustainable development and resilient urban design policies.

To establish enduring systems capable of withstanding urbanization and emerging risks, a balanced approach must be affected between financial limitations, technological progress, and community involvement^8,9^. Achieving resilience in the face of aging urban infrastructure and public concerns necessitates effective collaboration among various stakeholders. A robust urban society serves as a cornerstone of economic prosperity, offering access to markets, employment opportunities, and essential services.

Seismicity in Oman is primarily influenced by regional tectonic activity from the Makran Subduction Zone and Zagros Fold-Thrust Belt, causing occasional moderate tremors that highlight the need for proactive seismic risk assessment^10,11^. Muscat lies within a zone of low-to-moderate seismic activity, but its proximity to the Makran Subduction Zone and the Zagros Fault System in southern Iran makes it vulnerable to distant, yet impactful regional earthquakes^12^. These events (Table 1) have emphasized the need for urban seismic preparedness^13^, particularly for rapidly growing areas like Al-Seeb within the Muscat Governorate. Earthquakes around the Muscat Governorate have been reported with magnitudes below 7.5, for which the ${M}_{w}$ scale may not yield accurate size estimates. The ${M}_{w}$ scale has notable limitations: (1) a mathematical flaw below ${M}_{w}$ 7.5 due to the incorrect use of the Purcaru and Berckhemer^14^ equation, (2) validation restricted to Southern California, (3) poor agreement with ${m}_{b}$ and ${M}_{s}$ below ${M}_{w}$ 7.5^15,16^, and (4) systematic overestimation of radiated energy. To address these issues, the ${M}_{wg}$ scale proposed by Das et al.^17^ is employed in Table 1, as it avoids the mathematical flaw, aligns more closely with ${m}_{b}$ and ${M}_{s}$ across all magnitude ranges, and improves correspondence with radiated energy estimates.

**Table 1:** Historical seismicity around muscat governate.

Date	Magnitude (${{\varvec{M}}}_{{\varvec{w}}{\varvec{g}}}$)	Epicenter location
January 19, 2025	1.8	3 km south of Muscat City
November 30, 2024	1.6	Al Amerat (~ 8 km SW of Muscat)
January 14, 2024	5.1	Southeastern Iran
October 21, 2023	4.4	Offshore near Sur, Oman Sea
April 13, 2023	5.5	Owen Fracture Zone, Arabian Sea
July 2, 2022	5.8	Hormozgan, Iran
April 9, 2013	6.0	Bushehr, Iran
March 11, 2010	5.5	South Iran (near Qeshm)
November 28, 1945	8.1	Makran Subduction Zone

Al-Seeb stands out as a densely populated area hosting residential complexes, commercial offices, and tourist attractions. A comprehensive seismic hazard and geophysical testing-based site investigation, carried out by researchers affiliated with Earthquake Monitoring Center (EMC) at Sultan Qaboos University. The significant seismic risk inherent in Oman, particularly in the event of a potential earthquake scenarios has been studied^12,18–22^. In the interest of ensuring the safety and sustainability of urban locales like Al-Seeb, the implementation of robust, forward-looking urban planning and risk assessment strategies becomes imperative. Such strategies must draw extensively upon accurate and multidisciplinary urban modeling methodologies. The adoption of resilient urban planning practices necessitates significant advancements in both research and policy formulation.

In the present study, an integrated framework combining the analytic hierarchy process (AHP) and geographic information system (GIS) based spatial mapping techniques is employed to evaluate the earthquake vulnerability of urban residential structures in the Al-Seeb region through a multi-criteria decision making (MCDM) methodology^23^. This approach is crucial for Omani cities as it enables data-driven zoning, prioritizes retrofitting needs, and supports urban resilience planning in alignment with Oman Vision 2040. Specifically for the Al-Seeb region, the GIS-AHP model captures spatial heterogeneity by integrating localized factors such as soft soil conditions, rapid urban expansion, and non-engineered structures, thereby enabling precise microzonation and risk-informed urban development.

The assessment incorporated a comprehensive set of parameters including seismic, geotechnical, physical, structural, environmental and socioeconomic vulnerability indicators. The methodology is further extended to construct thematic seismic risk matrices and culminates in the generation of a detailed microzonation map delineating five distinct seismic risk zones, ranging from very low to very high. For selected towns within Al-Seeb, residential buildings are systematically classified into three damage categories: low, moderate, and high, based on their expected seismic performance. The outcomes of this study are well aligned with the United Nation’s Sustainable Development Goal (UN-SDG) 11, which underscores the importance of inclusive, safe, resilient, and sustainable urban environments. The methodological robustness of this study lies in its holistic, multi-criteria framework that integrates both structural and non-structural vulnerability dimensions. This comprehensive approach not only advances seismic risk assessment but also supports evidence-based policymaking for enhancing the resilience and sustainability of urban infrastructure.

## Literature review

Earthquakes and their associated hazards have profound direct and indirect impacts on human society, resulting in loss of life, property, and economic disruption. Past seismic events have demonstrated that intense ground motion can lead to varying degrees of building damage, ranging from minor structural issues to complete collapse, depending on site conditions and structural design parameters^24–28^. For instance, the 2001 Gujarat earthquake (${M}_{wg}$ 7.6) in India incurred an approximate total economic loss of USD 4600 million^29^. Similarly, the 2004 Indian Ocean earthquake (${M}_{wg}$ 9.4) triggered tsunamis that claimed the lives of approximately 0.25 million people across several countries, including India, Indonesia, Malaysia, Maldives, Myanmar, Sri Lanka, Seychelles, Thailand, and Somalia^30,31^. Inadequate resources and infrastructure hampered the timely delivery of emergency aid and medical assistance to affected communities in these regions. During the 1985 Mexico earthquake (${M}_{wg}$ 7.4), local site effects exacerbated secondary hazards such as landslides and ground subsidence, intensifying the earthquake’s impact^32–34^. The presence of soft soil conditions in Mexico City amplified seismic waves, leading to widespread building collapse and structural failure. Buildings located in regions characterized by high seismic activity or unstable ground conditions, such as proximity to fault lines or liquefaction-prone areas, inherently face greater susceptibility to earthquake damage, irrespective of their age or construction quality^35^. This vulnerability was evident during the 2005 Kashmir earthquake (${M}_{wg}$ 7.5), which inflicted significant damage on residential structures and associated infrastructure in India, Pakistan, and Afghanistan^21,36^.

The choice of construction materials and the principles of building engineering significantly influence the potential damage incurred during seismic events. Structures constructed with substandard materials or lacking engineered designs are particularly prone to catastrophic consequences. For example, during the 2010 Haiti earthquake (${M}_{wg}$ 6.8), the country’s poorly constructed buildings, many of which were not engineered to withstand seismic forces, experienced severe damage^4,37^. The resultant loss index amounted to USD 8.5 billion, leading to the complete disruption of critical infrastructure in urban environments. Hospitals and medical facilities were among the structures devastated, depriving many injured individuals of essential medical assistance. Moreover, socioeconomic factors such as lifestyle, employment patterns, and demographic characteristics also contribute to vulnerability^38^. Higher population densities often lead to the construction of numerous buildings in limited spaces, often without adequate consideration for seismic resilience. This was evident during the 2008 Wenchuan earthquake (${M}_{wg}$ 7.8), where densely populated urban environments faced shortages of essential supplies including food, water, shelter, and medical aid^39^.

The arrangement and coordination of utility lines such as gas, electricity, and water distribution systems play a crucial role in determining the extent of post-seismic hazards and subsequent economic losses^40,41^. Damage to gas lines, for instance, has been implicated in fires that pose significant threats to human life, as observed in seismic events like the 1989 Loma Prieta earthquake (${M}_{wg}$ 5.1), 1995 Kobe earthquake (${M}_{wg}$ 6.7), and 2011 Tohoku earthquake (${M}_{wg}$ 9.2). Similarly, disruptions to water supply lines, as witnessed during the 2010 Haiti earthquake (${M}_{wg}$ 6.8), result in widespread shortages of clean water for drinking, cooking, and sanitation^42^. This exacerbates the spread of waterborne diseases and complicates relief efforts and overall recovery endeavors. Damage to electricity infrastructure can impede transportation systems and result in economic losses due to business interruptions. Thus, swift restoration of electricity services post-earthquake is imperative for mitigating adverse impacts on human life.

The presence of essential facilities such as hospitals and fire stations are crucial for providing relief and medical assistance to the injured and affected individuals. Instances such as the 2010 Haiti earthquake (${M}_{wg}$ 6.8) and the 2005 Kashmir earthquake (${M}_{wg}$ 7.5) underscore the distressing consequences of inadequate medical resources, which contribute to high mortality rates^43^. Furthermore, the integrity of road networks and their connectivity to buildings in earthquake-affected areas is a critical consideration during urban planning. Damage or blockages to roads and highways, as evidenced in seismic events like the 1999 Chi-Chi earthquake (${M}_{wg}$ 7.6) and the 2008 Wenchuan earthquake (${M}_{wg}$ 7.8), can impede evacuation efforts, leaving individuals vulnerable to the seismic aftermath^44^.

Assessing risk solely based on a single vulnerability criterion is inadequate. It’s imperative to consider multiple criteria to gain a comprehensive understanding of the risks faced by urban residential buildings. Prior research conducted in various nations like South Korea, Iran, India, Malaysia, Mexico, China, Italy, Portugal, and the United States has leveraged the AHP, introduced by Saaty^45^, as a valuable multi-criteria decision-making technique. These studies integrated structural factors with socioeconomic factors such as population density and land use, as well as geotechnical factors. Physical factors such as proximity to hazardous facilities (e.g., gas stations) and access to vital amenities (e.g., hospitals, road networks) are also incorporated. Environmental factors, including distance to faults, rivers, per capita GDP, and normalized difference vegetation index (NDVI), have been integrated into vulnerability mapping^46^. The outcomes of such studies can be utilized in developing risk matrices^47^, grading building damage^48^ and assessing economic losses^49^.

Several regional seismic hazard studies in the Gulf region have evaluated tectonic settings^50–52^, active faults^53–55^, and ground motion characteristics^56,57^,^58^,^59^, particularly in seismically influenced areas such as southern Iran, northern UAE, and eastern Saudi Arabia. Previous studies in countries like Saudi Arabia, UAE, Iran, and Turkey have applied GIS-AHP approaches to integrate geological, geotechnical, and seismological parameters for seismic microzonation^60–64^. These efforts facilitated spatial vulnerability mapping and hazard classification of urban zones. However, many of these studies lacked inclusion of structural vulnerability indicators, exposure elements, and post-seismic recovery factors in the analysis. The present work on Al-Seeb region in Oman incorporates a comprehensive multi-criteria framework by integrating seismic hazard intensity, ground response parameters, building inventory, and social vulnerability layers, offering a more holistic evaluation compared to prior studies in Iran, Turkey, UAE, and Saudi Arabia.

Unlike the earlier studies by^12,19^ which primarily focused on hazard mapping and empirical ground motion data, the present study approach includes exposure, vulnerability functions, and urban planning insights, making it more suitable for resilience planning and emergency preparedness. This study also introduces a scalable, decision-support framework that bridges hazard data with urban vulnerability indicators, enabling tailored mitigation strategies. It can be extended to other Gulf cities, such as Abu Dhabi, Sohar, Dammam, and Ras Al Khaimah for improved seismic risk-informed urban development in line with UN-SDG 11 goals.

## Study area

**Fig. 1:**
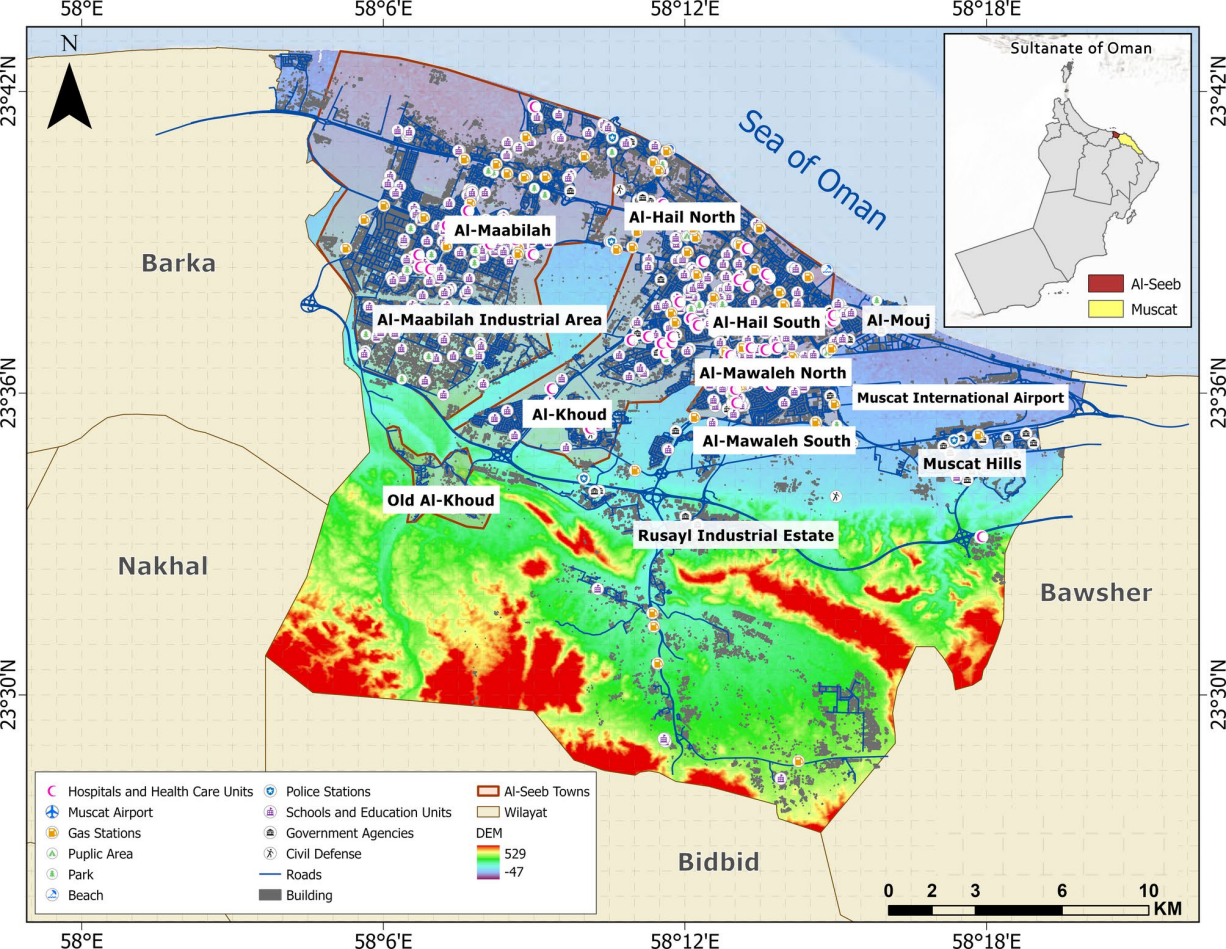
Geographic representation of the study area: Al-Seeb region, Muscat. The base map is quoted from the publicly available Google satellite imagery maps (https://maps.google.com/).

The governorate of Muscat encompasses a total geographic expanse of 3500 km^2^, subdivided into six administrative *wilayats*: Al-Amarat, Bawsher, Muscat (old town), Muttrah, Qurayyat, and Al-Seeb. Among these, Al-Seeb spans an area of 488.9 km^2^ and hosts the strategically significant Muscat International Airport on its north-eastern periphery. This airport serves as a critical transportation hub within the Sultanate of Oman, facilitating both domestic and international connectivity. Geographically, the northern boundary of Al-Seeb is delineated by its coastline, while the southern boundary extends toward the Rusayl industrial estate. Figure 1 provides a detailed representation of geographic layout of Al-Seeb, including the spatial distribution of dangerous and vital infrastructure facilities.

**Fig. 2:**
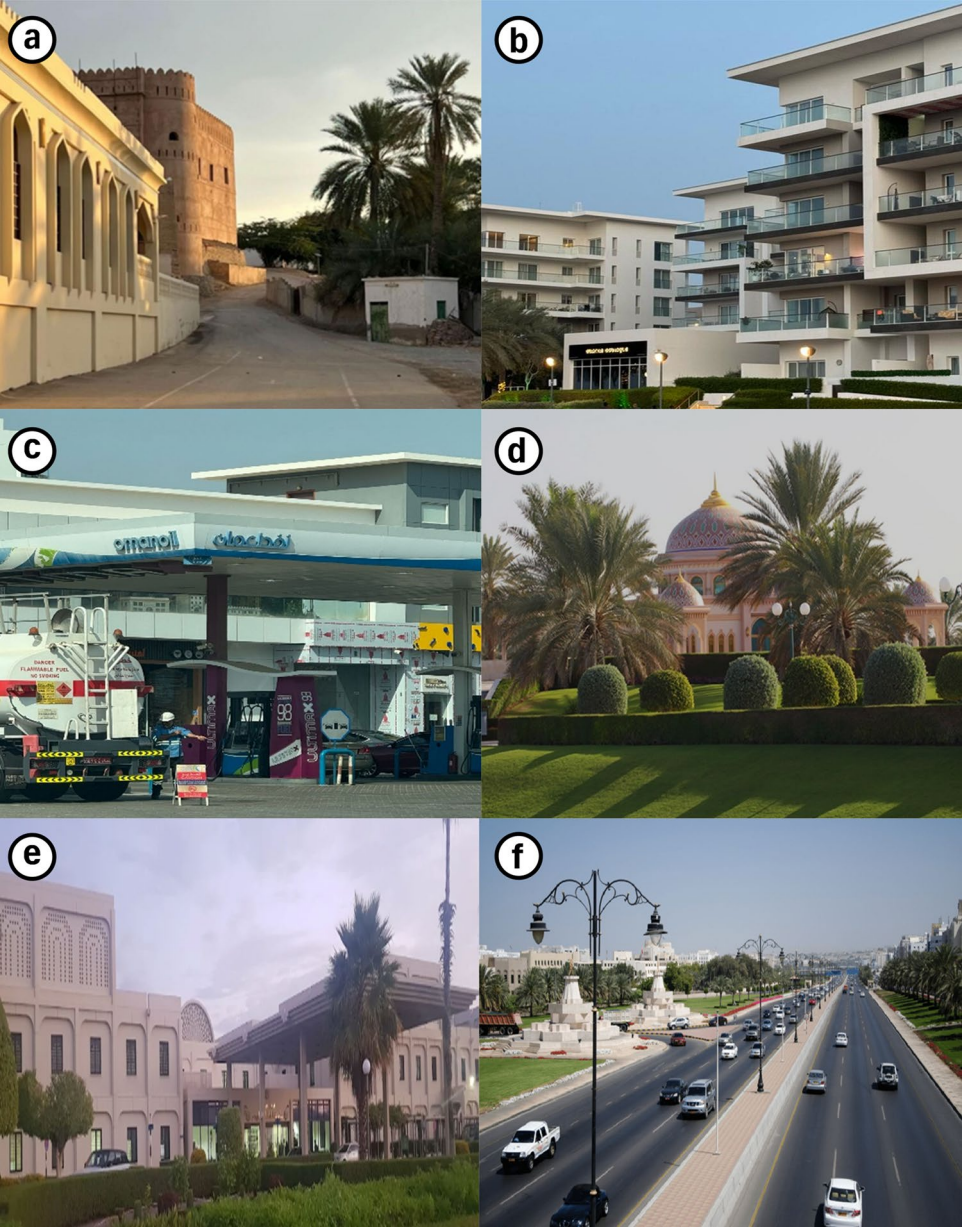
(**a**) Traditional residential zone in Al-Khoud; (**b**) modern housing development in Al-Mouj; (**c**) gas station situated in the densely populated urban area of Al-Mawaleh North; (**d**) Al-Sahwa Public Park to be used for disaster emergencies; (**e**) reinforced concrete (RC) building, Sultan Qaboos University Hospital and (**f**) primary transportation corridor, Sultan Qaboos road (Representative field and site photographs illustrating prevailing site conditions and structural typologies, captured by Hajar Al-Qayoudhi, for contextual support in vulnerability assessment).

Within the study area lies Old Al-Khoud (Fig. 2a), a historic settlement characterized by traditional Omani architectural heritage, with many structures dating back over a century. In contrast, newer developments in Al-Mouj (Fig. 2b) and Al-Hail North have emerged over the past fifteen years, showcasing modern construction techniques and advanced urban planning principles. The central region of Al-Seeb exhibited high population density and is predominantly urbanized, with key built-up areas including Al-Khoud and Al-Maabilah. The reliance on private transportation has necessitated the establishment of gas stations (Fig. 2c) at intervals of approximately one kilometer along major roadways. Healthcare infrastructure within Al-Seeb is concentrated in the central region, where the majority of hospitals and medical centers are located. A representative public park and a hospital building are illustrated in Figs. 2d and 2e, respectively. Sultan Qaboos road (Fig. 2f) serves as the primary arterial route, interconnecting various towns within Al-Seeb and providing seamless connectivity to other regions of the Muscat governorate. Table 2 presents the population distribution data for the study area, offering insights into demographic patterns across Al-Seeb.

**Table 2:** Population of Al-Seeb by gender and nationality up to June-2024^65^.

Nationality	Gender		Total
	**Male**	**Female**	
Omani	136,571	135,348	271,919
Foreigner	190,009	81,495	271,504
Total	326,580	216,843	543,423

## Methodology

**Fig. 3:**
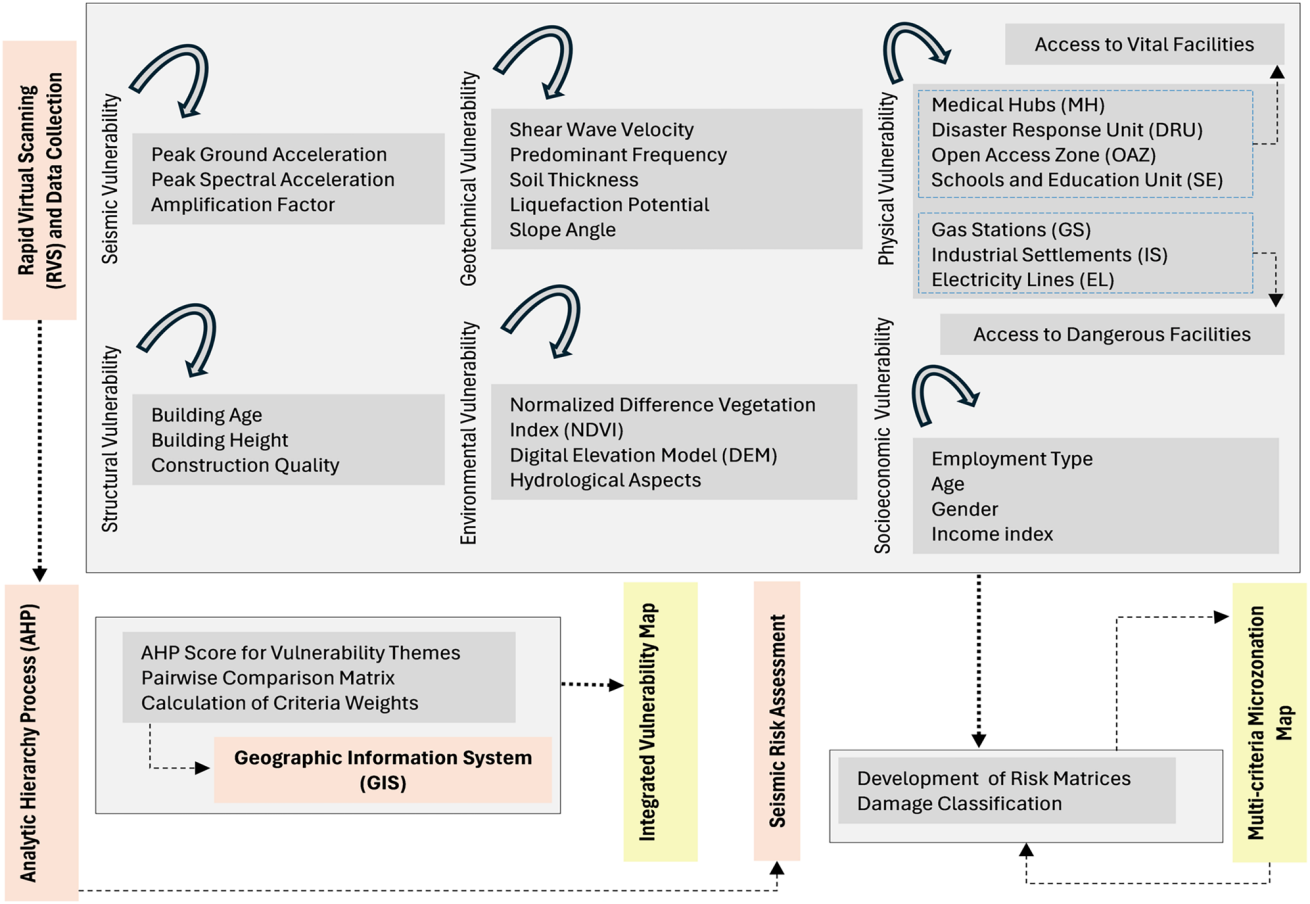
Schematic flowchart depicting the stepwise approach for generating the microzonation map.

The sophisticated design of urban residential buildings and the advancement of contemporary projects necessitate substantial funding at local, regional, and national levels across various stages of development^5^. However, safeguarding these structures and reducing costs in the event of natural disasters present a persistent challenge. Addressing a single vulnerable parameter is insufficient, as historical precedents have demonstrated. To accomplish this, the present study is structured into three principal phases, each addressing a critical component of the analysis. The sequential methodology adopted is visually represented in Fig. 3, which outlines the systematic workflow underpinning the present work.

### Rapid visual scanning (RVS) survey and data collection

The preliminary step entailed the systematic acquisition of multi-source data corresponding to the selected vulnerability indicators, as summarized in Table 3. RVS survey was implemented (February 2025 to May 2025) across key residential zones within the Al-Seeb, wherein structural attributes such as building height, construction year, and qualitative indicators of construction integrity were documented.

**Table 3:** Selected vulnerability themes in the present study.

Vulnerability themes	Sub-themes	Data source	Data resolution	Update cycle	Spatial accuracy
Seismic	Peak ground acceleration	OSC 2013, EMC-D	Zonation map (1:250,000)	Fixed (2013), periodic EMC updates	Moderate (site classification)
	Peak spectral acceleration		Zonation map (1:250,000)	Fixed (2013), periodic EMC updates	Moderate
Geotechnical	Shear wave velocity	EMC-D, GTC	Site-specific (10 to 30 m depth)	Project-based	High (GPS-verified)
	Fundamental frequency		Site-specific (microtremor)	Project-based	High (± 20 m)
	Soil thickness		Site-specific (borehole/MASW)	Project-based	High
	Liquefaction potential		GIS layer (1:50,000 to 1:100,000)	As per soil investigations	Low
	Slope angle		DEM-based (10 to 30 m grid)	On-demand	Moderate
Physical (access to vital facilities)	Medical hubs (MH)	RVS survey, G-Earth	Point locations	Periodically updated	High (± 10 to 30 m)
	Disaster response unit (DRU)		Point locations	Periodically updated	High
	Open access zone (OAZ)		Polygon (site boundary)	Project-specific	High
	Schools and education unit (SE)		Point locations	Periodically updated	High
Physical (access to dangerous facilities)	Gas stations (GS)	RVS survey, G-Earth	Point locations	Periodically updated	High
	Industrial settlements (IS)		Polygon or cluster area	Periodically updated	Moderate to high
	Electricity lines (EL)		Line features	Periodically updated	Moderate (satellite trace)
Structural	Building age	RVS survey, NCSI	Attribute data	Census-linked (10 years)	High
	Building height		Attribute data	Census-linked or direct survey	High
	Construction quality		Qualitative (grade scale)	Project-specific	Moderate
Environmental	Normalized difference vegetation index (NDVI)	G-Earth	30 m resolution (Landsat)	Monthly to seasonal	Moderate to high
	Digital elevation model (DEM)		10 to 30 m grid	Static	Moderate
	Hydrological aspects		Watershed or catchment (1:50,000)	Case-specific	Moderate
Socioeconomic	Employment type	RVS survey, NCSI	Block or ward level	Census-linked	Moderate to high
	Age		Block or ward level	Census-linked	Moderate to high
	Gender		Block or ward level	Census-linked	Moderate to high
	Income index		Block or ward level	Census-linked	Moderate

* Here, OSC 2013 = Oman seismic design code for buildings 2013 edition; EMC-D = Earthquake Monitoring Center depository; GTC = geotechnical consultancies; RVS survey = rapid visual screening survey; G-Earth = Google Earth and NCSI = National Center for Statistical Information, Muscat (Sultanate of Oman).

In the context of Al-Seeb, the use of satellite-derived or interpolated data faces challenges due to dense urban fabric, complex land cover, and coastal interference, which may reduce the reliability of parameters like NDVI, soil thickness, and slope. Such limitations may lead to inaccuracies in capturing localized variations in ground conditions and infrastructure distribution, thereby affecting the precision of seismic vulnerability mapping in the area.

### Multi-criteria vulnerability assessment

The data set belonging to all vulnerability themes described in Table 3 were integrated into a hierarchical framework, with appropriate weights assigned to each theme and ranks to the sub-theme within each primary theme, following the AHP introduced by Saaty^45^. AHP, a widely adopted MCDM approach, incorporates both objective and subjective factors to aid decision-makers in disaster management. AHP can be summarized as follows:

#### Define the vulnerability theme and sub-themes

The first step in the AHP involves defining the main vulnerability theme along with its associated sub-themes. These themes represent the key factors contributing to vulnerability in a given context and are typically identified through expert judgment, field observations, and literature review. This hierarchical structuring of the decision problem lays the foundation for comparative analysis. For example, Table 3 provides a thematic breakdown used for assessing seismic vulnerability.

#### Conduct pairwise comparisons and assign weights

Once the themes and sub-themes are established, they are systematically compared in a pairwise manner to determine their relative importance. This is done using a scale proposed by Saaty^66^, where values range from 1 (equally important) to 9 (extremely more important). Alternatives are compared using specific criteria (${x}_{1}$, ${x}_{2}$… ${x}_{n}$). Scores are scaled between 1 and 9, reflecting criteria from equally important to extremely important, based on expert opinions and/or available data. Criteria weights (${w}_{1}$….${w}_{n}$) are evaluated using the normalized matrix under the condition that the sum of weights equals one ($\sum_{j=1}^{n}{W}_{j}=1$). The weighted sum vector is formulated as:

1 $${W}_{i}=\sum {x}_{ij}{w}_{j}$$

where ${x}_{ij}$ indicates the ${i}^{th}$ class rank for the ${j}^{th}$ layer, and ${W}_{j}$ is the weight for the ${j}^{th}$ layer.

Table 4 presents the assumed weightages and ranking factors assigned to the six selected vulnerability themes.

**Table 4:** Weightage criteria assumed and consistency ratio ($CR$) for selected vulnerability themes in the present study.

Vulnerability themes	Sub-themes	Weights	Rank	Overall weight	Normalized rank	Consistency ratio (${\varvec{C}}{\varvec{R}}$)	Weight justification
Seismic	Peak ground acceleration	0.14	1	0.14	0.50	0.013	Reflects the direct contribution of ground motion parameters to damage potential; crucial for hazard exposure
	Peak spectral acceleration		2	0.28	1.00		
Geotechnical	Shear wave velocity	0.13	1	0.13	0.20	0.025	Important for site response and ground failure potential; includes shear wave velocity, soil conditions that influence local hazard
	Fundamental frequency		2	0.26	0.40		
	Soil thickness		3	0.39	0.60		
	Liquefaction potential		4	0.52	0.80		
	Slope angle		5	0.65	1.00		
Physical (access to vital facilities)	Medical hubs (MH)	0.23	1	0.23	0.25	0.008	High priority due to the critical role of medical hubs, schools, and disaster response units in post-earthquake emergency services and community recovery
	Disaster response unit (DRU)		2	0.46	0.50		
	Open access zone (OAZ)		3	0.69	0.75		
	Schools and education unit (SE)		4	0.92	1.00		
Physical (access to dangerous facilities)	Gas stations (GS)	0.23	1	0.11	0.33	0.009	Equally weighted with vital facilities due to the potential for secondary hazards from industrial zones, gas stations, and electrical infrastructure during seismic events
	Industrial settlements (IS)		2	0.22	0.66		
	Electricity lines (EL)		3	0.33	1.00		
Structural	Building age	0.11	1	0.11	0.33	0.018	Building-related factors such as age, height, and construction quality directly affect structural performance and collapse risk during earthquakes
	Building height		2	0.22	0.66		
	Construction quality		3	0.33	1.00		
Environmental	Normalized difference vegetation index (NDVI)	0.07	1	0.07	0.33	0.032	Least direct impact on immediate seismic damage; includes NDVI, DEM, and hydrological aspects, which are more relevant for secondary hazards and accessibility
	Digital elevation model (DEM)		2	0.14	0.66		
	Hydrological aspects		3	0.21	1.00		
Socioeconomic	Employment type	0.09	1	0.09	0.25	0.012	Moderate influence, representing the capacity of communities to prepare for and recover from seismic events; includes employment, income, age, and gender factors
	Age		2	0.18	0.50		
	Gender		3	0.27	0.75		
	Income index		4	0.36	1.00		

#### Check for consistency in judgments

The final step in the AHP method focused on verifying the consistency of the judgments made during the pairwise comparison process. Ensuring logical consistency is essential to maintain the credibility of the resulting weights. To evaluate this, the consistency index ($CI$) is computed and compared to a random index ($RI$) as defined by Saaty^45^ an Saaty^67^. The resulting consistency ratio ($CR$) provides a measure of reliability, as presented in Eq. (3). A $CR$ value of 0 indicates complete consistency, while values exceeding 0.1 signal unacceptable inconsistency, requiring the pairwise comparison process to be repeated for improved accuracy and confidence in the derived weights.

2 $$CI= \frac{{\beta }_{max}-OD}{OD-1}$$

3 $$CR= \frac{CI}{RI}$$

In Eq. (2), ${\beta }_{max}$ denotes the principal (largest) eigenvalue of the pairwise comparison matrix, while $OD$ represents the order (dimension) of the matrix.

In this study, spatial analysis was conducted for each grid by partitioning the entire study area into uniform grid cells of 1.0 km × 1.0 km. Within the mapping platform, normalized rank values, detailed in Table 4 were integrated with corresponding empirical data associated with each specific vulnerability theme. This process facilitated the generation of individual thematic maps, each representing the spatial distribution of a distinct vulnerability dimension.

### Multi-criteria risk assessment

To perform a comprehensive seismic risk analysis, distinct vulnerability themes were selected to capture critical risk dimensions. The qualitative attributes of each thematic map were evaluated for individual buildings identified through the RVS survey. Raster-based seismic and geotechnical vulnerability indices were spatially averaged at the building level and subsequently ranked according to classification criteria detailed in Table 4.

The quantile classification method was adopted to ensure a uniform distribution, with each vulnerability grade encompassing approximately 20% of the dataset. This facilitated the construction of thematic risk matrices consistent with the^68^ risk consequence framework. These matrices were then employed to generate a microzonation map delineating seismic risk zones in Al-Seeb into five categories: very low, low, moderate, high, and very high.

In the final stage, this microzonation output was integrated with categorical and structural attributes from the RVS data to classify buildings into three damage states: minor, moderate, and extensive^69^ methodology was adapted to the local context by integrating site-specific seismic demand parameters, such as peak ground acceleration and peak spectral acceleration and local amplification factors, with structural attributes from RVS survey. Seismic capacity was estimated using lateral strength ratios, fundamental period approximations, and ductility-based performance levels for dominant typologies in Al-Seeb, such as URM and RC frames. Damage thresholds were defined in terms of inter story drift ratios and capacity-demand ratios: minor damage was assigned for drift ratios < 0.2% and demand-to-capacity ratio (DCR) < 0.7, moderate damage for drift between 0.2–0.5% and DCR between 0.7–1.0, and extensive damage for drift > 0.5% or DCR > 1.0. This engineering-driven adaptation provided realistic seismic damage classification tailored to Oman’s built environment.

## Multi-criteria vulnerability assessment

### Seismic vulnerability assessment

**Fig. 4:**
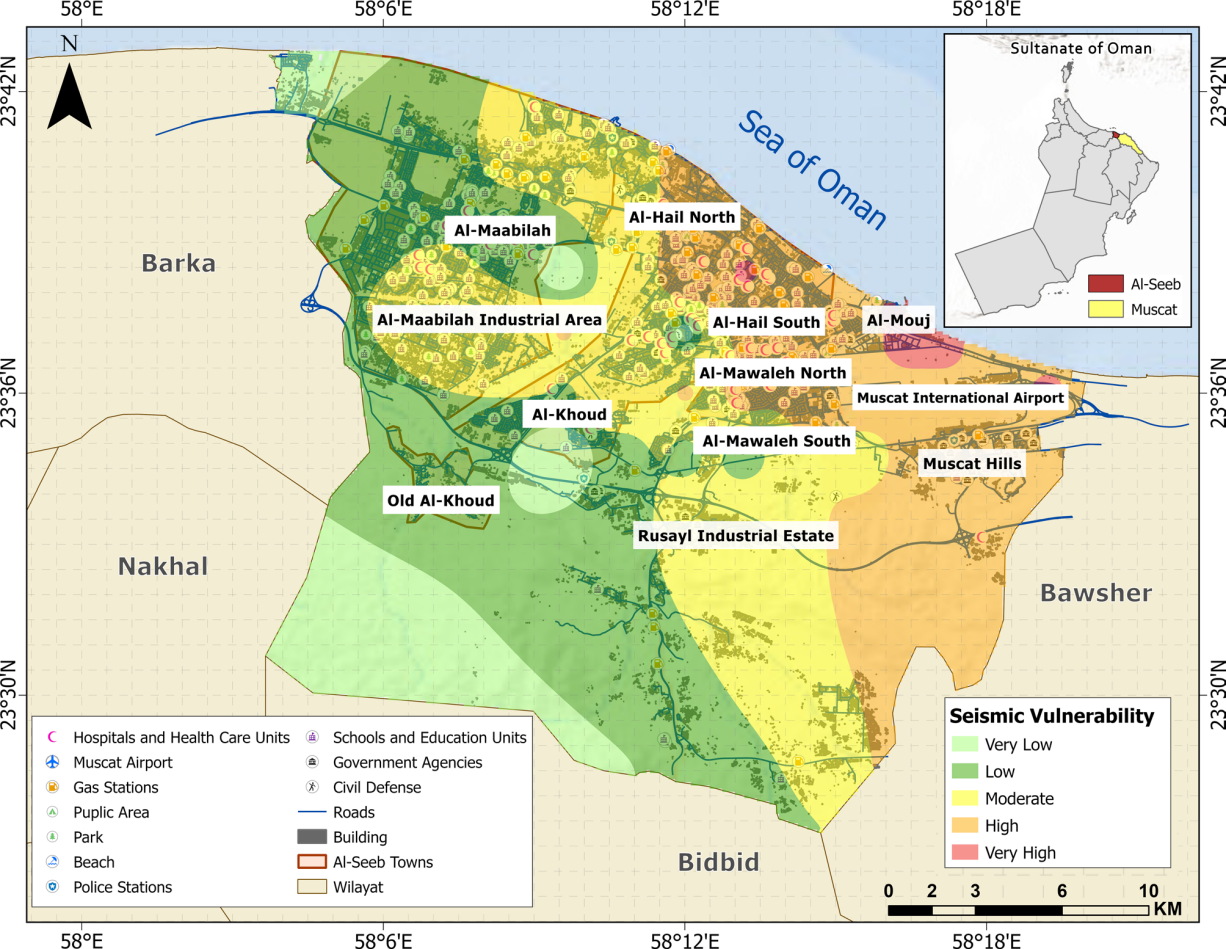
Seismic vulnerability mapping based on earthquake-related parameters. The base map is quoted from the publicly available Google satellite imagery maps (https://maps.google.com/).

The seismic vulnerability map shown in Fig. 4 delineates spatial variations in potential earthquake-induced impacts by integrating key seismic parameters for return period of 475 and 2475 years. The map reveals a progressive increase in vulnerability from the southwestern zone, characterized by very low intensity, toward the northeastern region, transitioning through low, moderate, and high vulnerability levels. Notably, localized zones of extended vulnerability are observed near the Al-Seeb shoreline.

### Geotechnical vulnerability assessment

In Al-Seeb, the majority of sites exhibited shear wave velocity values between 550 m/s and 765 m/s, whereas isolated locations in Al-Hail recorded values below 400 m/s, indicating relatively weaker subsurface conditions. Fundamental frequencies in Al-Seeb ranged from 12.25 Hz to 21.5 Hz; however, sites proximal to the shoreline exhibited frequencies below 2.5 Hz, suggesting resonance with longer-period seismic waves. Soil thickness in the central zone averages approximately 9.5 m. Slope angles were extracted via spatial analysis using a high-resolution digital elevation model (DEM), as depicted in Fig. 1.

**Fig. 5:**
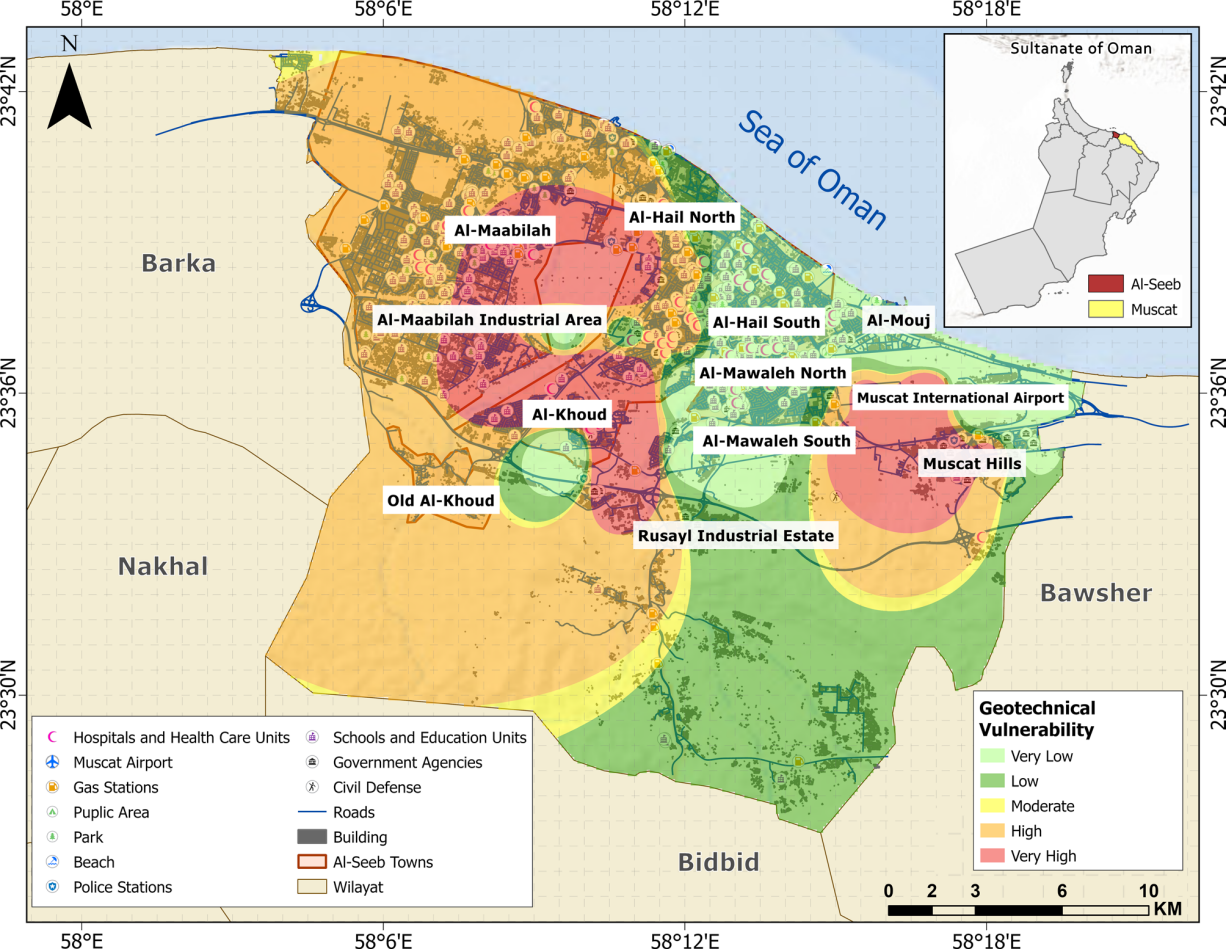
Geotechnical vulnerability assessment map. The base map is quoted from the publicly available Google satellite imagery maps (https://maps.google.com/).

The selected geotechnical indicators were integrated to generate a comprehensive geotechnical vulnerability map, shown in Fig. 5. The western segment of Al-Seeb demonstrated high vulnerability, while the eastern portion exhibited low to very low vulnerability. Nonetheless, isolated zones of very high geotechnical vulnerability can be observed in specific areas such as Al-Khoud, segments of Al-Maabilah (west), and the Muscat Hills region (east).

### Physical vulnerability assessment

**Fig. 6:**
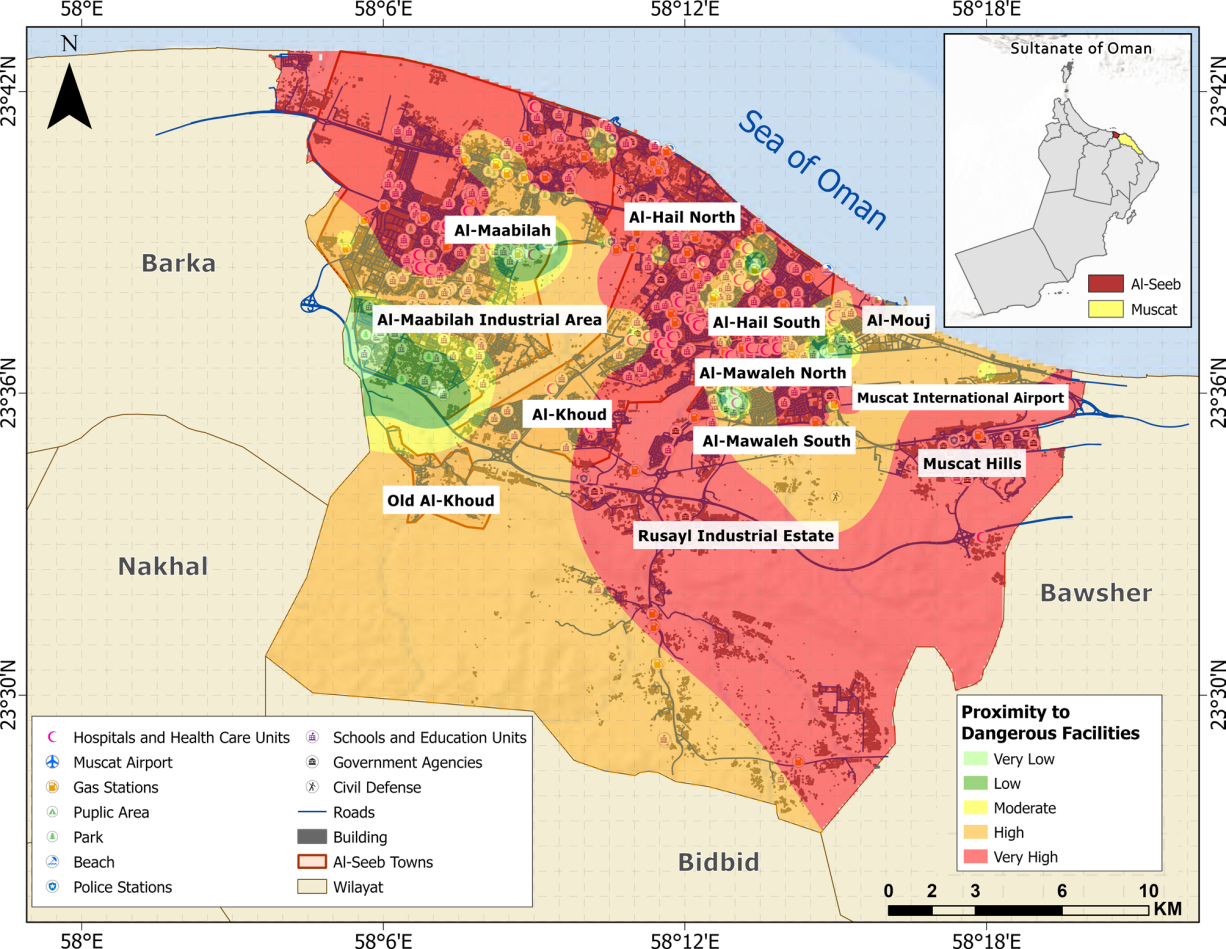
Vulnerability distribution concerning proximity to dangerous facilities. The base map is quoted from the publicly available Google satellite imagery maps (https://maps.google.com/).

Vulnerability assessment based on physical parameters encompasses two key aspects: proximity to dangerous facilities and proximity to vital facilities. In this study, gas stations, industrial establishments and electricity lines are classified as dangerous facilities. Figure 6 delineates spatial vulnerability zones based on site proximity to gas stations, adjacent industrial facilities, underground electricity cables (UEC) and overhead electricity cables (OEC). The vulnerability assessment integrates multiple parameters, including radial distance from gas stations, degree of site exposure (open access), connectivity to nearest road networks, and proximity to emergency response infrastructure such as fire stations and civil defense units. The vulnerability gradient across the map predominantly indicated critical exposure levels, particularly in northern and southeastern areas.

The Rusayl Industrial Zone, extending northwest toward Al-Maabilah, exhibited notably intensified hazard proximity due to the spatial concentration of industrial installations and gas stations near densely populated zones. However, response capacity remain inconsistent, as reflected by the variable distances to vital facilities.

**Fig. 7:**
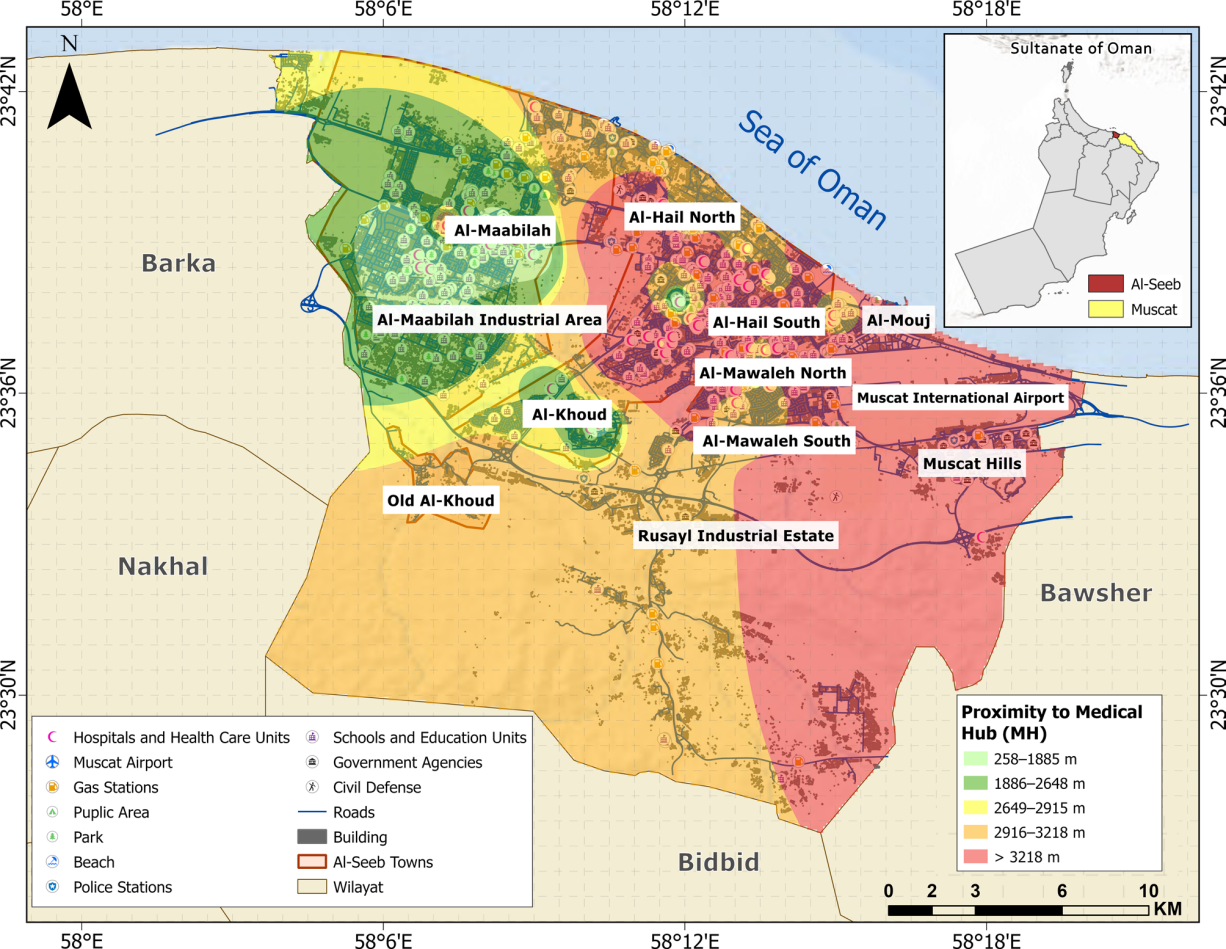
Vulnerability distribution concerning proximity to medical hubs (MH). The base map is quoted from the publicly available Google satellite imagery maps (https://maps.google.com/).

**Fig. 8:**
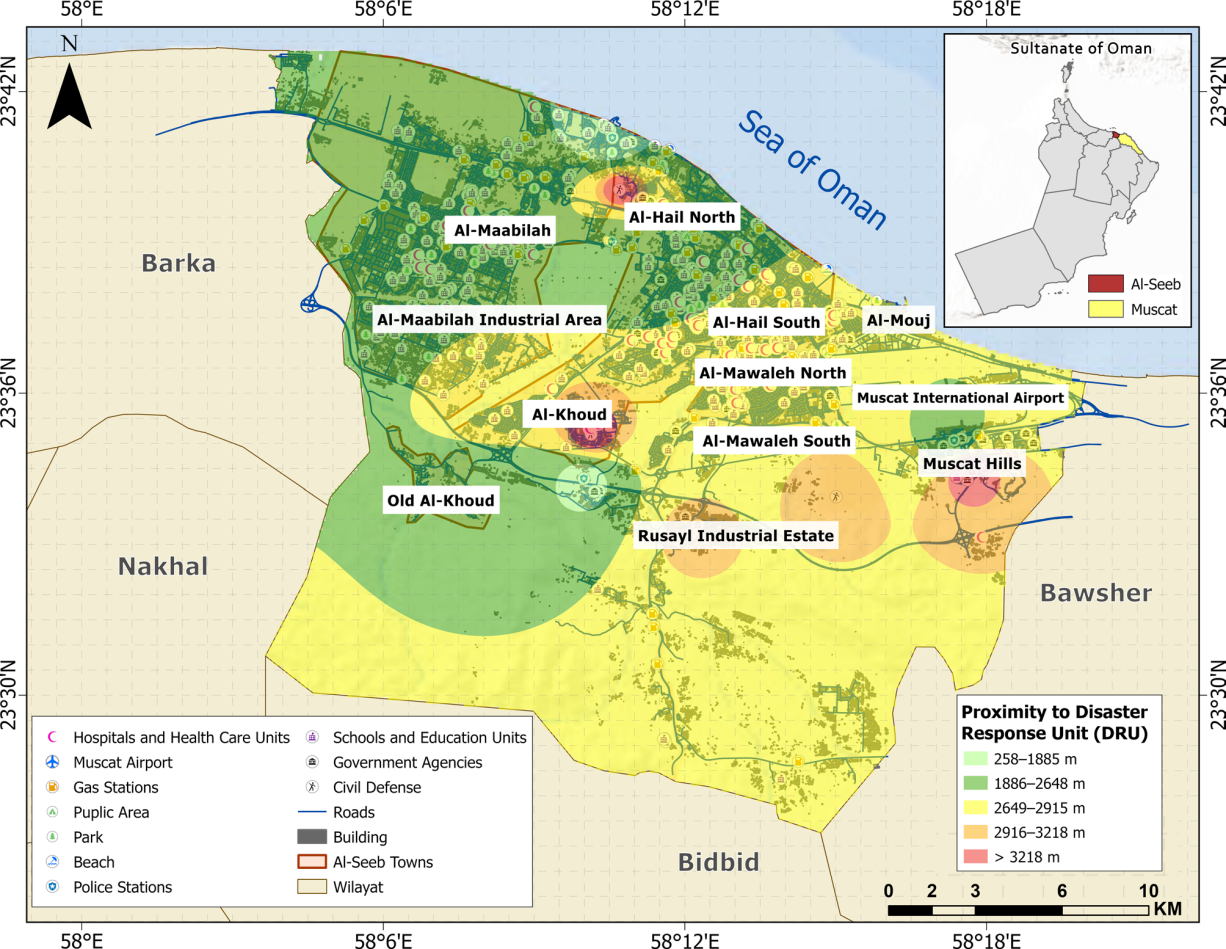
Vulnerability distribution concerning proximity to disaster response units (DRU). The base map is quoted from the publicly available Google satellite imagery maps (https://maps.google.com/).

**Fig. 9:**
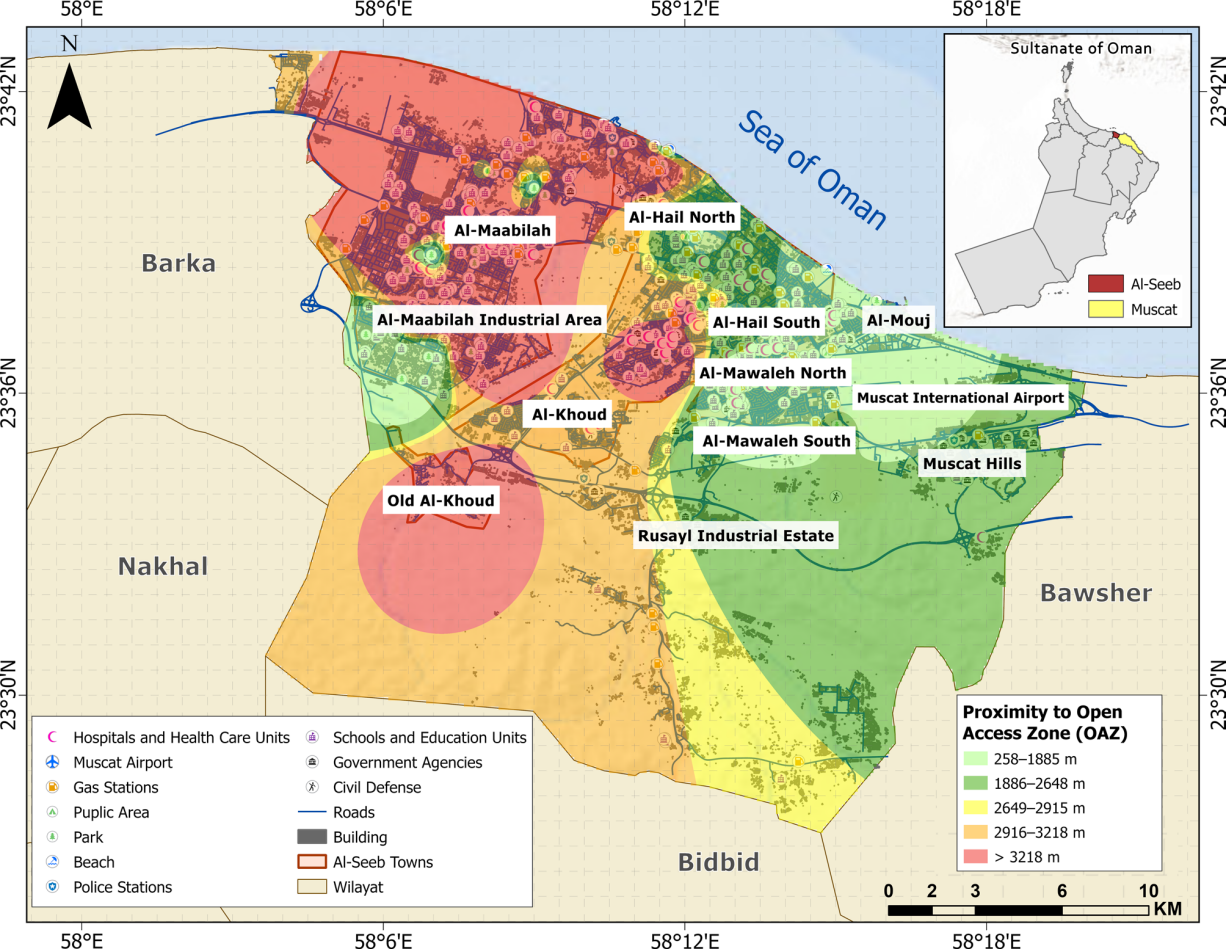
Vulnerability distribution concerning proximity to open access zones (OAZ). The base map is quoted from the publicly available Google satellite imagery maps (https://maps.google.com/).

**Fig. 10:**
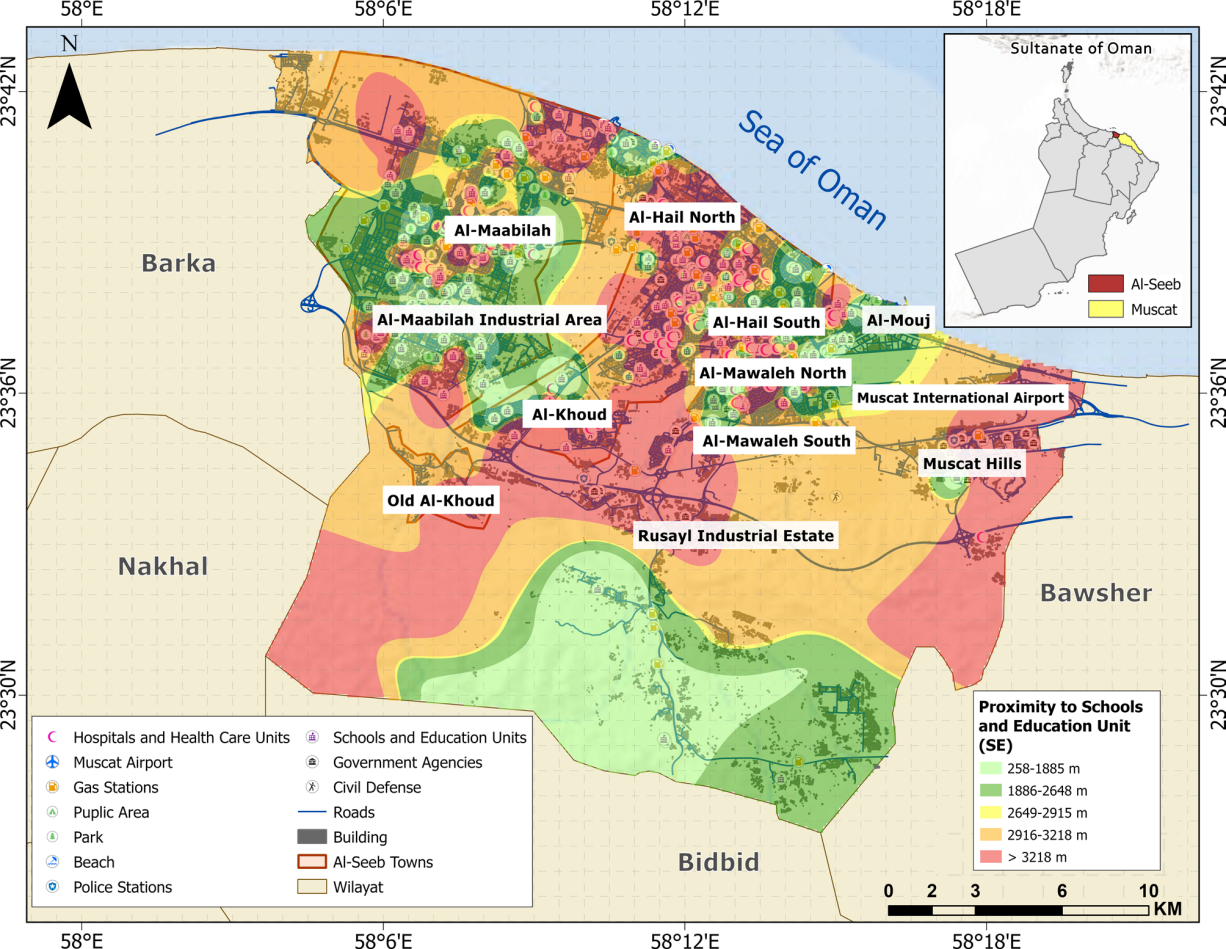
Vulnerability distribution concerning proximity to schools and educational institutions (SE). The base map is quoted from the publicly available Google satellite imagery maps (https://maps.google.com/).

**Fig. 11:**
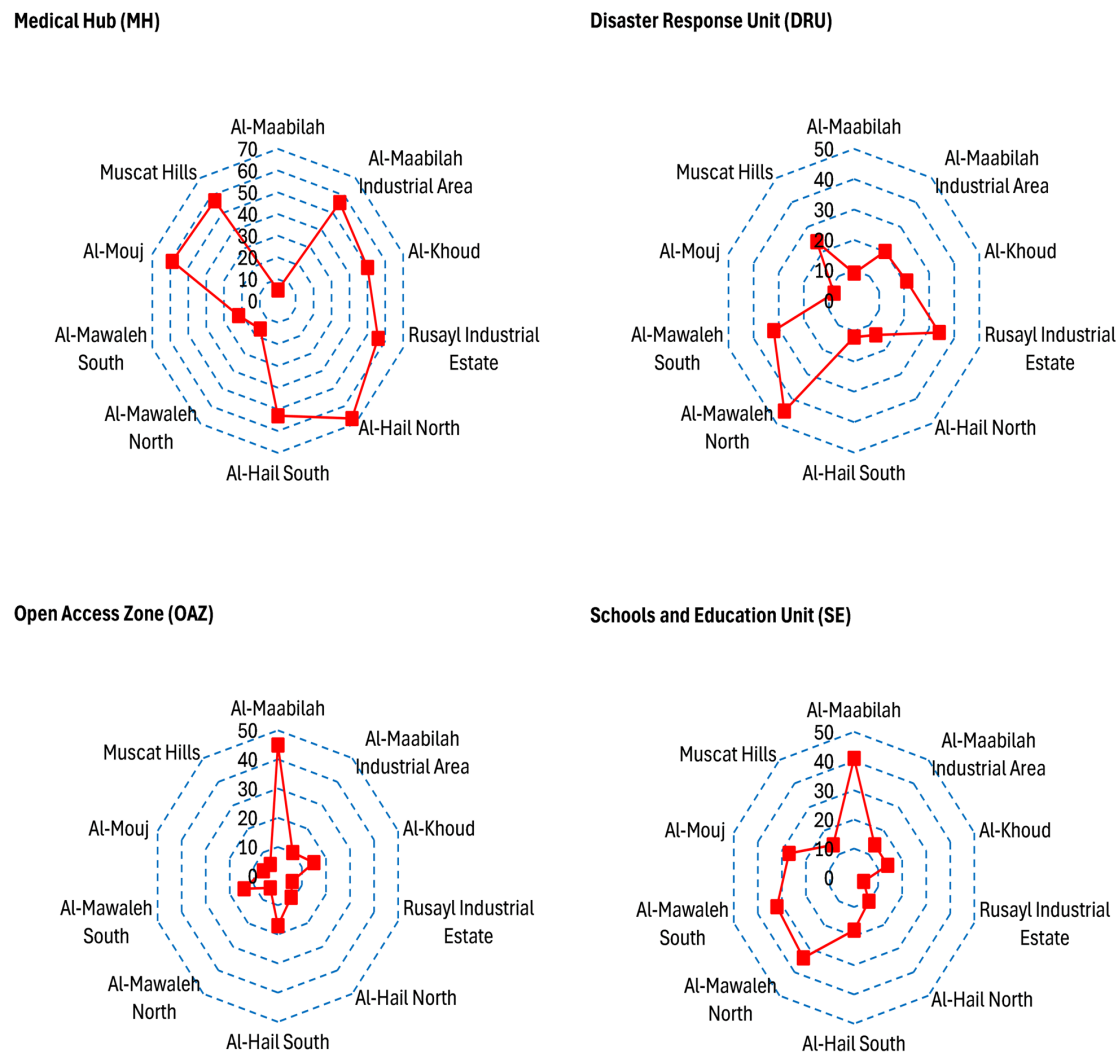
Analysis of sub-themes influencing accessibility to vital facilities in selected zones of Al-Seeb. The base map is quoted from the publicly available Google satellite imagery maps (https://maps.google.com/).

Medical hubs (MH), disaster response units (DRU), open access zones (OAZ), and schools and educational institutions (SE) are categorized as vital facilities (Figs. 7, 8, 9 and 10). Figure 11 provides analysis of sub-themes influencing accessibility to vital facilities in selected zones of Al-Seeb, with a focus on four types of facilities: medical hubs (MH), disaster response units (DRU), open access zones (OAZ), and schools and education units (SE). Al-Seeb is classified into ten zones, including Al-Maabilah, Al-Maabilah industrial area, Al-Khoud, Rusayl industrial estate, Muscat Hills, Al-Mouj, Al-Mawaleh North, Al-Mawaleh South, Al-Hail North and Al-Hail South. Notably, zones such as Al-Maabilah industrial area and Muscat Hills consistently demonstrated high scores across most sub-themes for all facility types, reflecting strong infrastructure and resource allocation.

In contrast, Al-Mawaleh North, Al-Mawaleh South, Al-Hail North and Al-Hail South exhibited persistently low scores, indicating significant challenges in accessibility. For instance, in the case of MH and DRU, these low-performing zones showed limited access, which could compromise emergency healthcare and disaster resilience. Similarly, accessibility to OAZ and SE is also constrained, suggesting a need for improved public spaces and educational infrastructure in these areas.

Overall, the analysis highlighted disparities in resource distribution and infrastructure development, emphasizing the need for targeted interventions. To address these gaps, recommendations include upgrading transportation systems, investing in facility construction in underserved zones, and implementing policies that ensure equitable resource allocation. By leveraging data-driven planning and community engagement, policymakers can work toward reducing accessibility disparities and enhancing the overall quality of life across Al-Seeb.

**Fig. 12:**
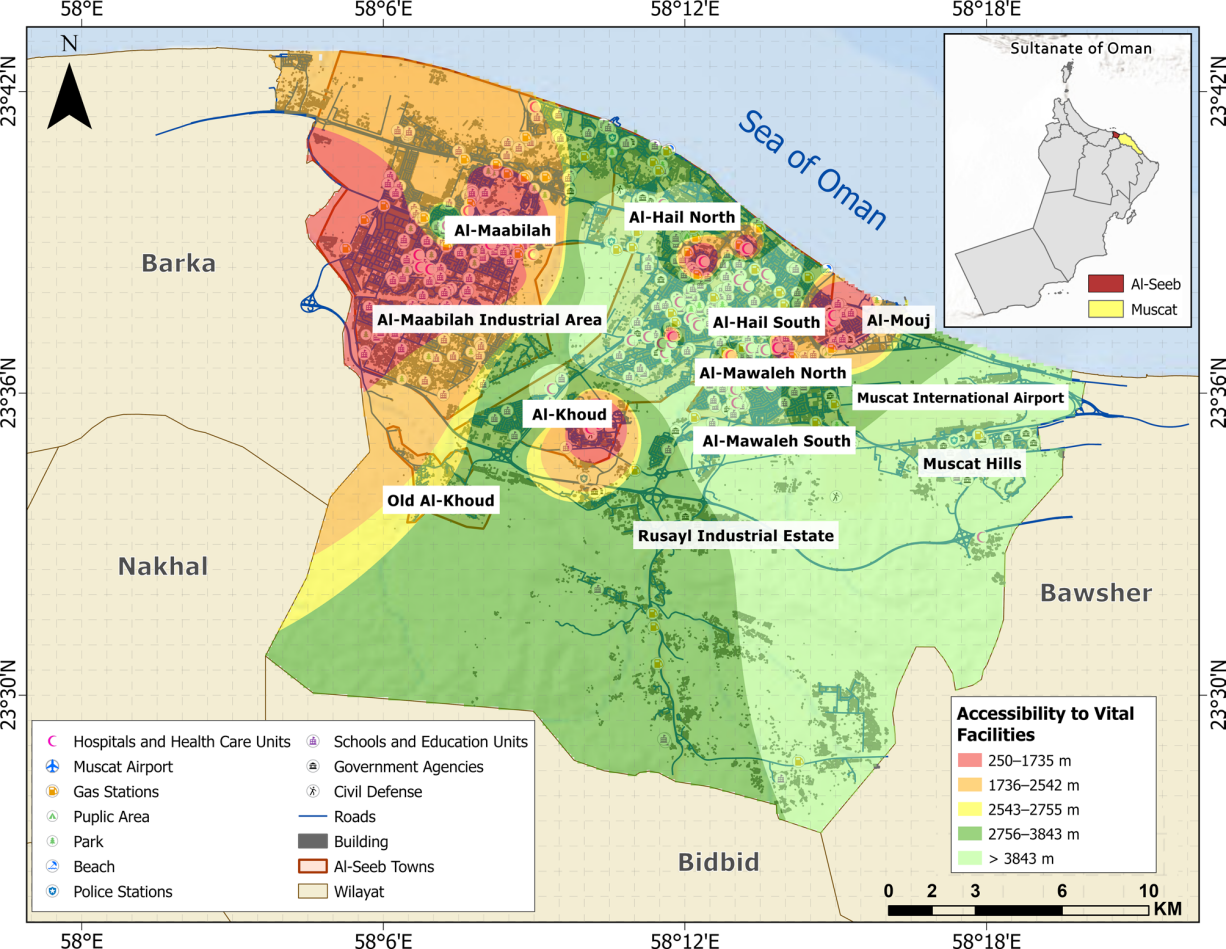
Vulnerability distribution concerning accessibility to vital facilities. The base map is quoted from the publicly available Google satellite imagery maps (https://maps.google.com/).

The integration of selected sub-themes culminated in the generation of a physical vulnerability map with respect to proximity to vital facilities, as illustrated in Fig. 12. Over half of the study area is represented by a green color code, signifying accessibility to essential services within an approximate range of 2756–3843 m. The Al-Maabilah industrial area is delineated in red, indicating the greatest spatial detachment from vital facilities, thereby reflecting a heightened level of physical vulnerability in terms of emergency accessibility and service coverage.

**Fig. 13:**
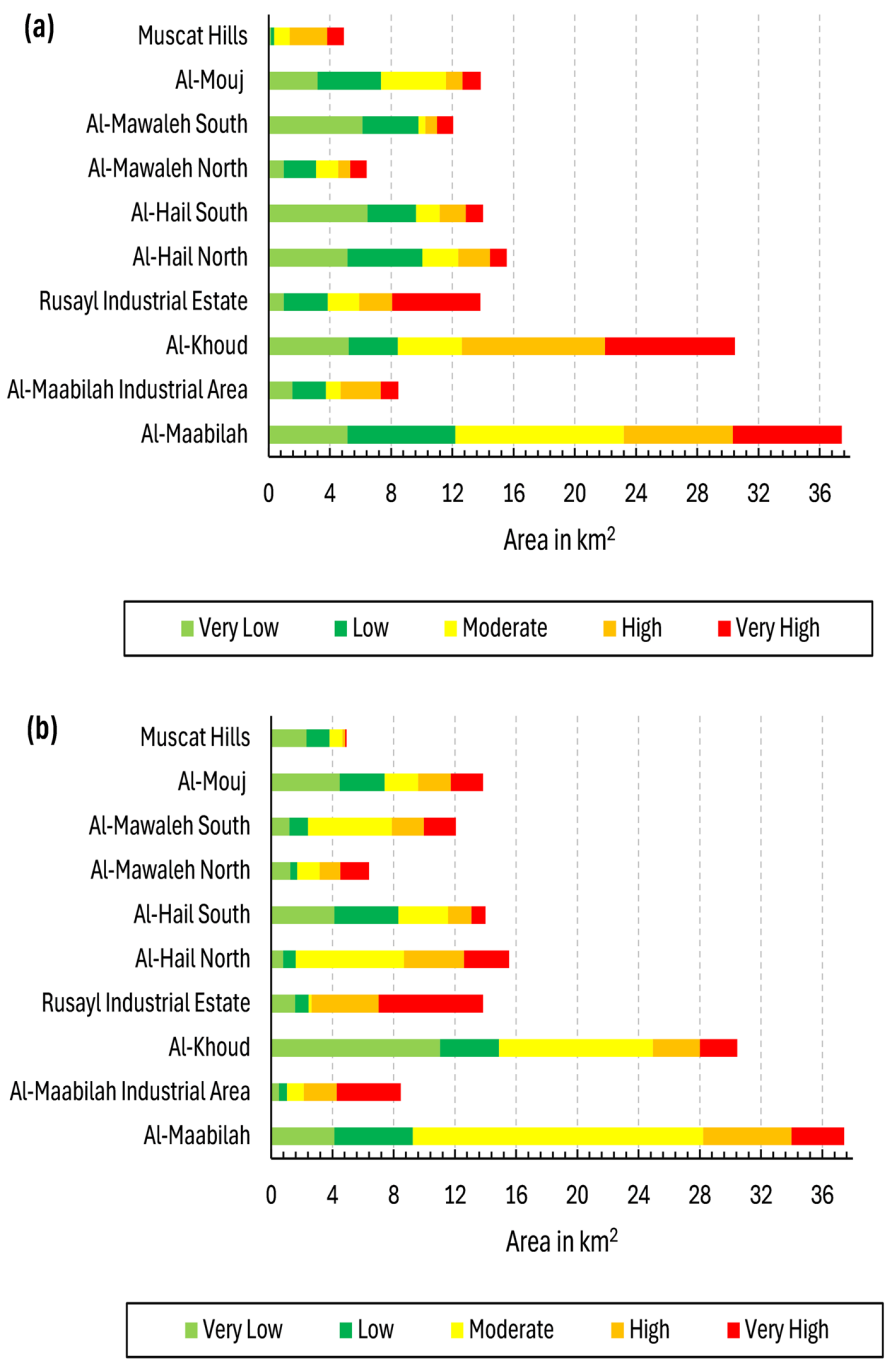
Spatial distribution of vulnerability levels across selected themes: (**a**) geotechnical vulnerability and (**b**) physical vulnerability.

A comparative analysis of the geotechnical and physical themes reveals distinct patterns in the distribution of vulnerability levels, reflecting the influence of both geological conditions and built-environment characteristics (Fig. 13). In terms of geotechnical vulnerability, zones such as Al-Khoud and Al-Maabilah industrial area exhibited high and very high vulnerabilities, with approximately 16 km^2^ and 27 km^2^, respectively. These findings suggested that these zones are highly susceptible to geotechnical hazards, likely due to unfavorable soil conditions, topography, or seismic potential.

Conversely, Muscat Hills demonstrated remarkable resilience, with nearly all its area (4 km^2^) classified under low vulnerability class, underscoring the stabilizing influence. Intermediate zones like Al-Hail North and Rusayl industrial estate showed a more balanced distribution, with significant portions (5 to 10 km^2^) in the moderate to high categories, indicating moderate geotechnical vulnerability.

When examining physical vulnerability, the trends largely aligned with geotechnical vulnerability but also highlighted additional factors influenced by urbanization and infrastructure quality. For instance, Al-Maabilah industrial area again stood out with ~ 9 km^2^ classified as very high, suggesting that its dense industrial infrastructure exacerbated physical vulnerability beyond geotechnical constraints. Similarly, Al-Khoud exhibited ~ 7 km^2^ in very high category, emphasizing the compounding effects of poor structural resilience. In contrast, Muscat Hills remained the least vulnerable zone, with negligible areas under high or very high physical vulnerability, likely due to its well-planned development and robust building design. Zones like Al-Hail South and Al-Mouj displayed moderate physical vulnerability, with ~ 3–5 km^2^ in the high category, reflecting a mix of stable geological conditions but varying degrees of infrastructural adequacy.

Comparatively, the impact of geotechnical vulnerability appears more deterministic, as it is primarily governed by inherent geological and geomorphological factors that are difficult to mitigate without significant engineering interventions. On the other hand, physical vulnerability is more dynamic and influenced by human activities, such as urban planning, construction practices, and disaster preparedness measures. For example, while Al-Khoud and Al-Maabilah industrial area faced severe challenges in both themes, the pronounced physical vulnerability in these zones suggested that anthropogenic factors further amplified their vulnerability profiles. In contrast, Muscat Hills benefited from favorable geotechnical conditions and superior physical infrastructure, resulting in consistently low vulnerability across both themes.

### Structural vulnerability assessment

**Fig. 14:**
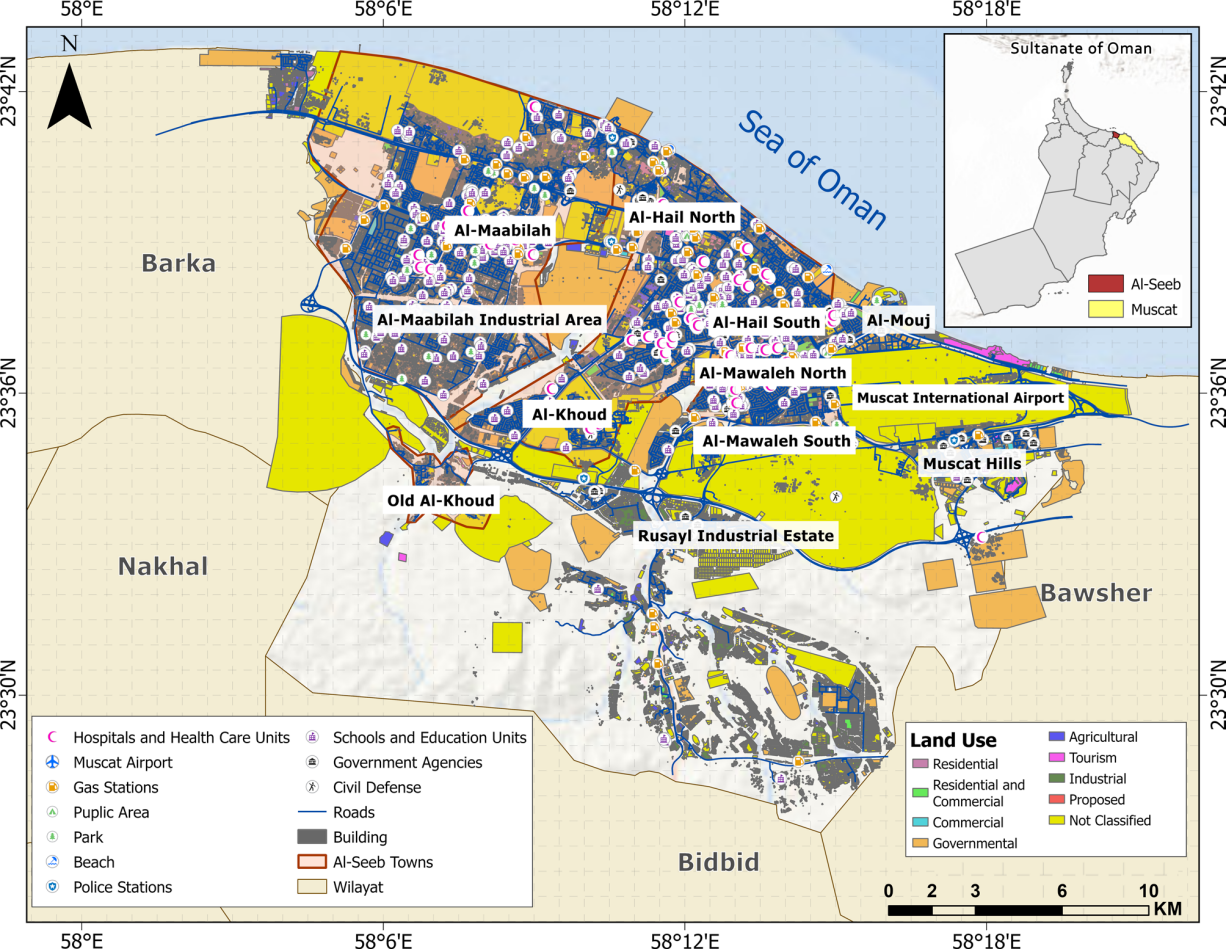
Urban planning map illustrating diverse land use zones and functional areas across the study area. The base map is quoted from the publicly available Google satellite imagery maps (https://maps.google.com/).

**Fig. 15:**
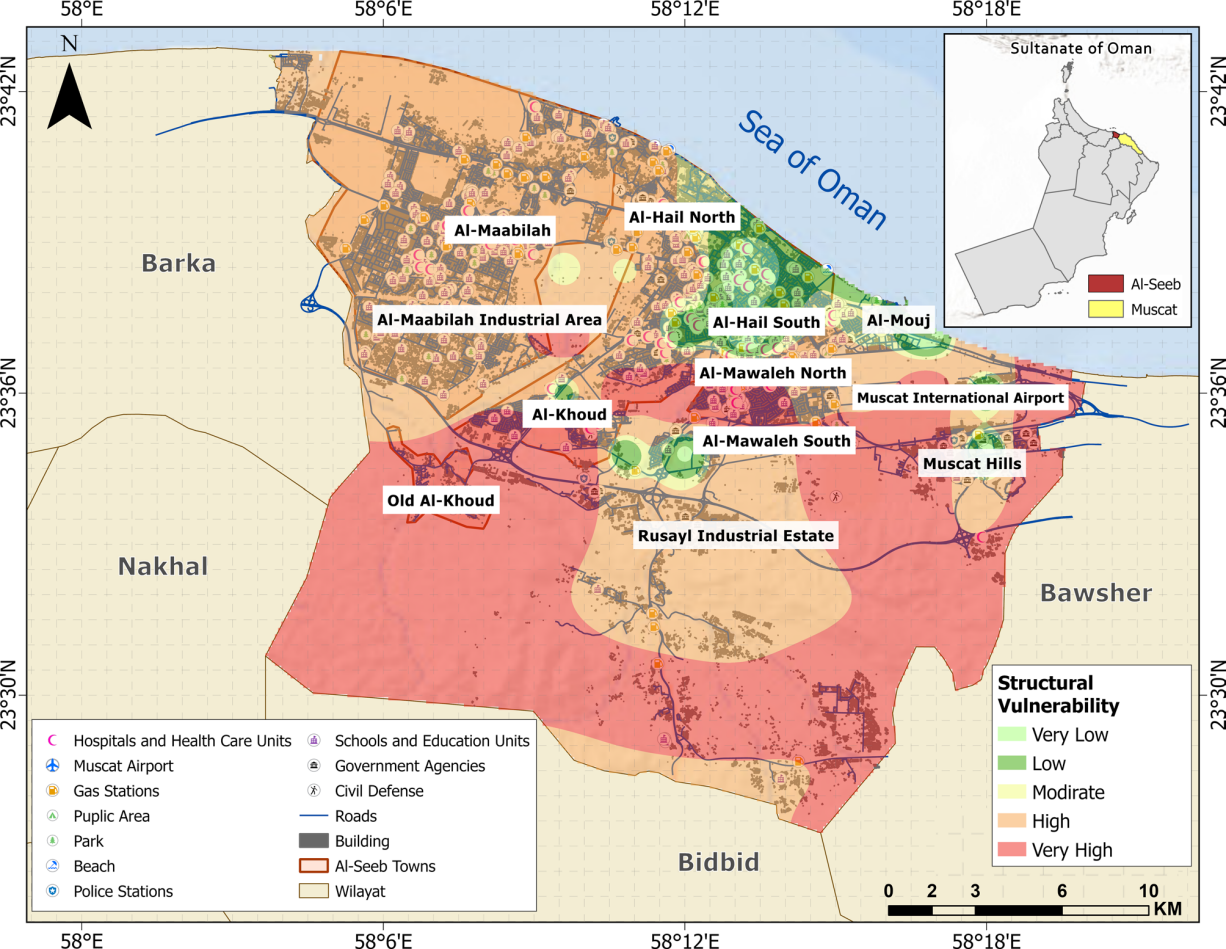
Structural vulnerability assessment map. The base map is quoted from the publicly available Google satellite imagery maps (https://maps.google.com/).

Based on data collected during RVS survey and NCSI^65^ data directory, building types and their uses are highlighted in the map shown in Fig. 14. It provides a clear classification of different types of buildings, highlighting the functional diversity of the urban landscape in Al-Seeb. It distinguishes between residential structures, commercial buildings, industrial and agricultural facilities. Certain areas also indicate specialized buildings related to government operations and tourism infrastructure. This categorization reflects a well-organized approach to urban planning, ensuring that each building type serves its intended purpose within a structured and integrated environment. Figure 15 provides a clear spatial representation of structural vulnerability across different urban areas.

Newer developments such as those found in Al-Mouj and parts of Al-Hail are shown in green, reflecting lower structural vulnerability. These areas consist mostly of mid to high rise buildings built with modern construction techniques and materials. Industrial zones like Rusayl and Al-Maabilah showed high vulnerability, depending on the age and maintenance of facilities. The variation in vulnerability across the map underscored the importance of targeted urban planning and infrastructure upgrades, particularly in historically developed or industrialized areas where structural weaknesses are more pronounced.

### Environmental vulnerability assessment

**Fig. 16:**
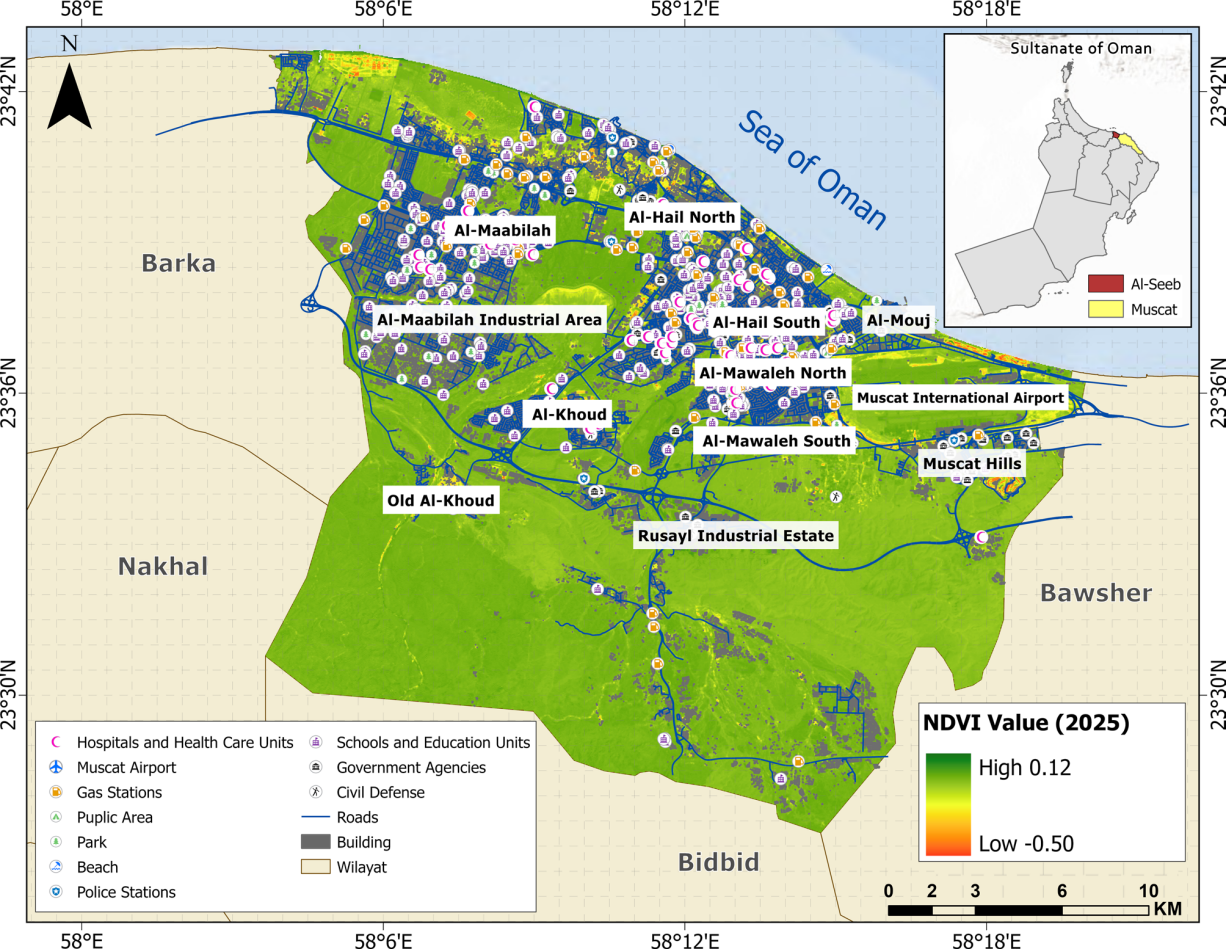
Normalized difference vegetation index (NDVI) map illustrating the spatial distribution of greenness levels throughout the study area. The base map is quoted from the publicly available Google satellite imagery maps (https://maps.google.com/).

The environmental vulnerability across the study area is highlighted by variations in vegetation cover derived from NDVI values, as illustrated in Fig. 16. Regions with high NDVI (dark green), such as parts of Al-Khoud and Old Al-Khoud, indicate dense and healthy vegetation, contributing to lower environmental vulnerability due to better soil stability, microclimate regulation, and biodiversity support. In contrast, low NDVI zones (yellow and orange), particularly in coastal areas reflect sparse vegetation and increased vulnerability to erosion, desertification, and climate-related stresses. Industrial and rapidly urbanizing areas show mixed patterns, where development pressures may be reducing natural land cover, further exposing these zones to environmental vulnerabilities.

**Fig. 17:**
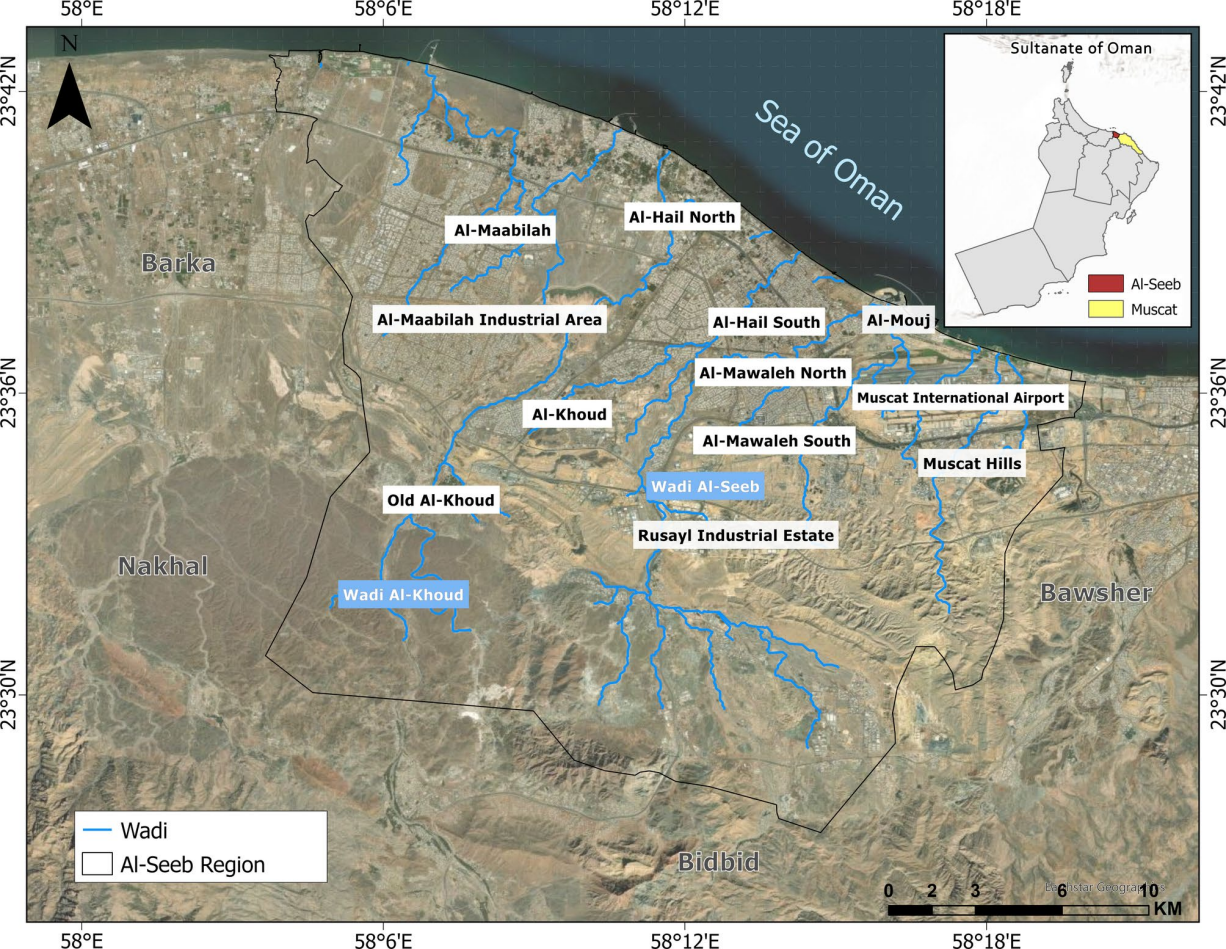
*Wadi* network in study area highlighting natural drainage paths. The base map is quoted from the publicly available Google satellite imagery maps (https://maps.google.com/).

The map shown in Fig. 17 illustrates a network of *wadis*, which act as natural conduits for water flow during rainfall events. These *wadis* are crucial for managing surface runoff and preventing localized flooding^70^. However, their effectiveness is compromised by urban encroachment, particularly in densely populated areas like Al-Hail North and South, Al-Maabilah industrial area and Old Al-Khoud. As these regions expand, natural *wadi* channels are often modified or obstructed, reducing their capacity to drain water efficiently. The *wadi* network is important for sustainable urban planning because it helps control floods, supports water recharge, and protects the environment in areas with low rainfall but occasional heavy storms.

### Socioeconomic vulnerability assessment

**Fig. 18:**
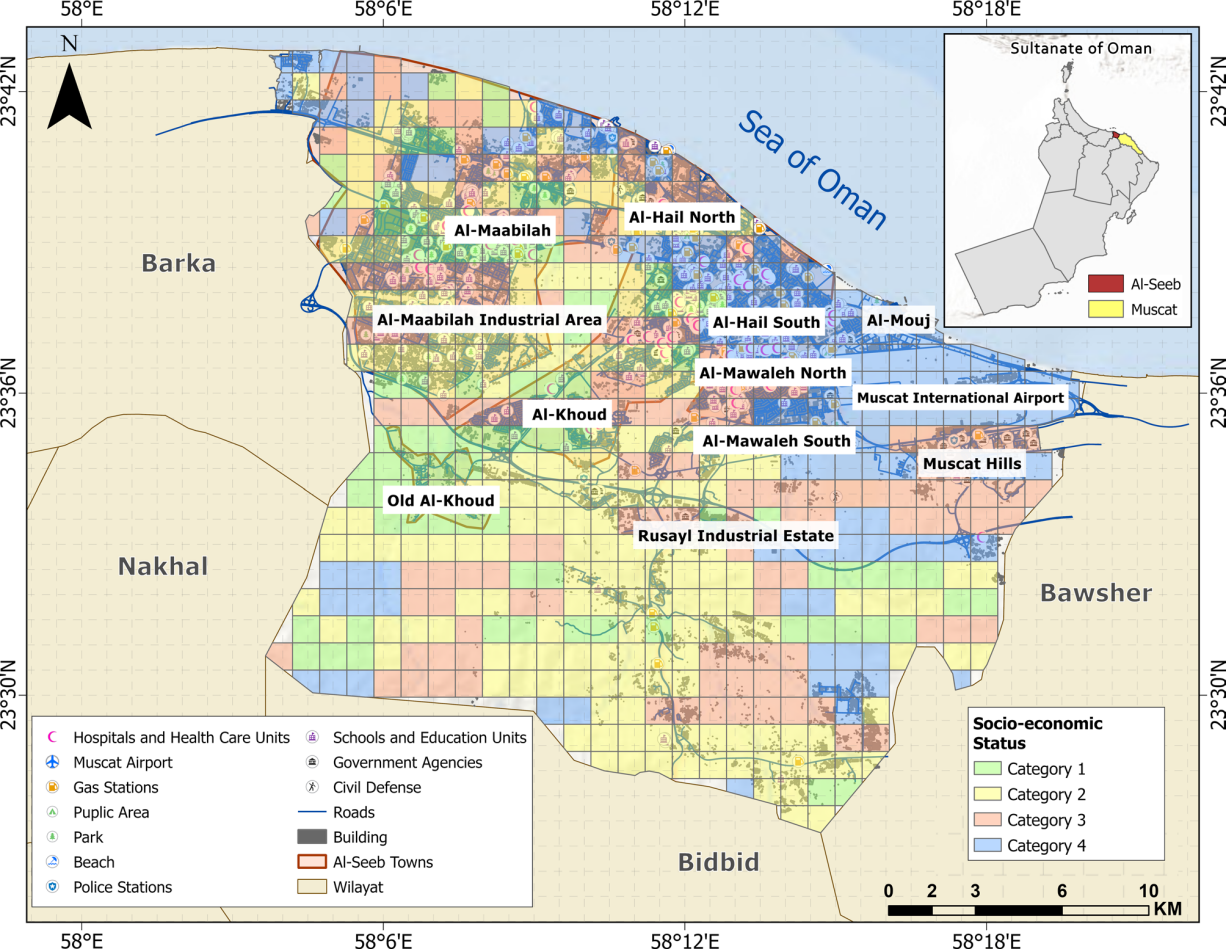
Spatial variation in socioeconomic status reflecting demographic profile and income level. The base map is quoted from the publicly available Google satellite imagery maps (https://maps.google.com/).

The Al-Seeb region of Muscat features a diverse socioeconomic profile, with a mix of middle and lower income households residing across its urban and suburban neighborhoods^65^. While some areas like Muscat Hills are characterized by higher living standards, others, such as Al-Mawaleh and Old Al-Khoud, reflect modest incomes with limited access to modern infrastructure. Most residents of the Al-Seeb region are employed in public services, retail, small businesses, and industrial sectors, reflecting a mixed occupational profile. Socioeconomic vulnerability is assessed using employment type, age, gender, and income index, categorizing the study area into four levels, with 1 indicating the least privileged and 4 representing the most well-off (Fig. 18).

Table 5 presents a normalized distribution of vulnerability themes across several areas in Al-Seeb, with each theme’s contribution summing to 100% per locality. It highlights that structural and geotechnical vulnerabilities consistently dominate across most areas, while physical access to hazardous facilities is more pronounced in industrial zones like Al-Maabilah Industrial and Rusayl.

**Table 5:** Normalized percentage distribution of vulnerability themes for selected areas in Al-Seeb region, based on relative influence derived from GIS-AHP overlay analysis.

Area	Vulnerability themes						
	**Seismic**	**Geotechnical**	**Physical (vital facilities)**	**Physical (dangerous facilities)**	**Structural**	**Environmental**	**Socioeconomic**
Al-Hail North	13	18	14	10	20	12	13
Old Al-Khoud	14	16	12	8	24	13	13
Al-Mawaleh North	13	17	13	10	22	12	13
Al-Khoud	14	16	12	9	23	13	13
Al-Maabilah	15	17	11	10	24	12	11
Al-Maabilah industrial area	16	18	10	14	25	9	8
Al-Mawaleh South	13	17	15	9	22	12	12
Al-Hail South	12	17	14	9	20	14	14
Muscat Hills	10	15	20	6	18	16	15
Rusayl Industrial estate	17	18	10	16	24	9	6
Al-Hail North	13	18	14	10	20	12	13
Overall Al-Seeb	35	42	28	18	47	32	29

**Fig. 19:**
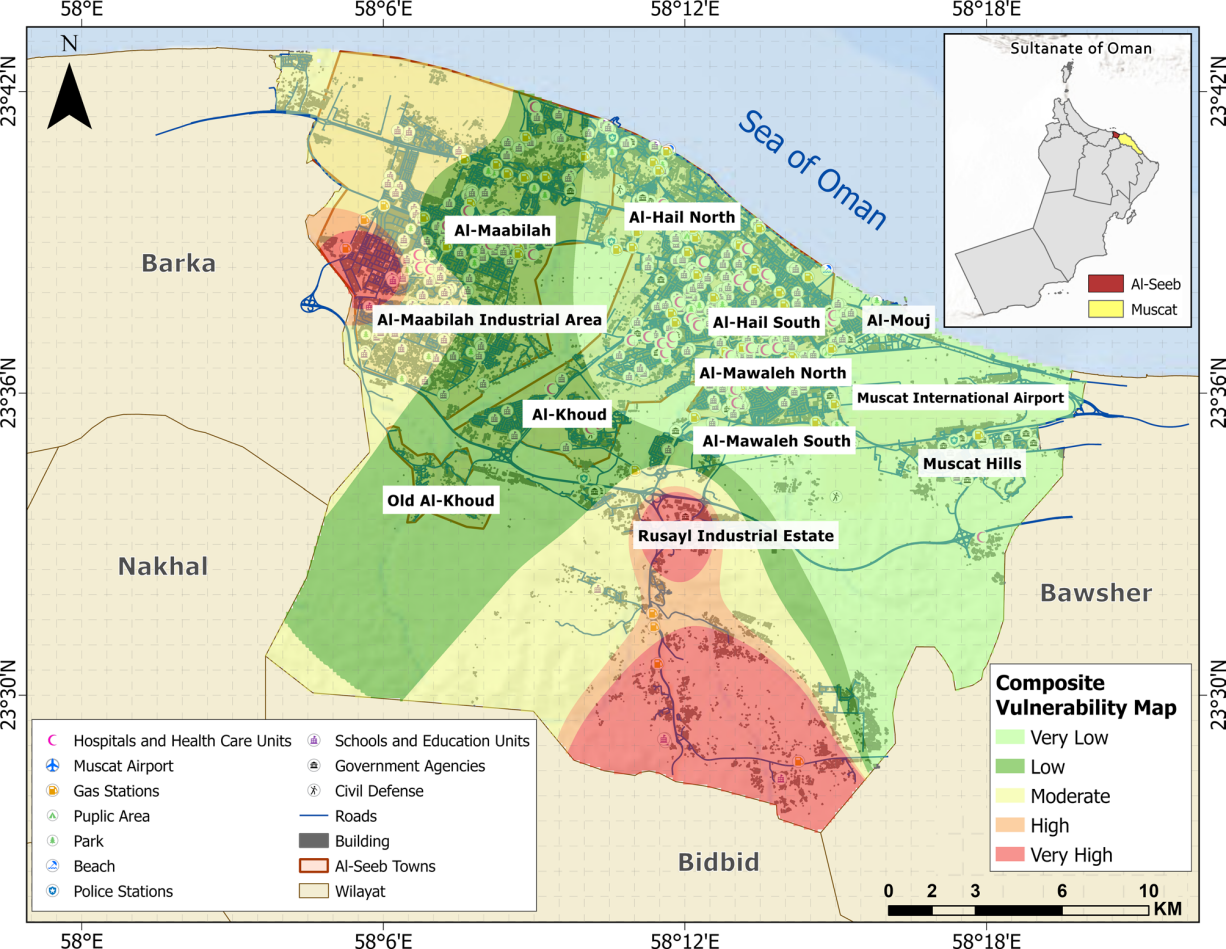
Composite vulnerability map showing the combined impact of all selected themes in the present study. The base map is quoted from the publicly available Google satellite imagery maps (https://maps.google.com/).

In the final phase, a composite vulnerability map (Fig. 19) is provided which facilitates more accurate identification of integrated vulnerable areas, enabling targeted mitigation strategies and informed decision-making for sustainable urban resilience. The map data can guide policymakers in prioritizing safer land use, infrastructure investment, and zoning regulations for future urban expansion in Al-Seeb or other Gulf cities by identifying area-specific vulnerability concentrations.

## Multi-criteria risk assessment

**Fig. 20:**
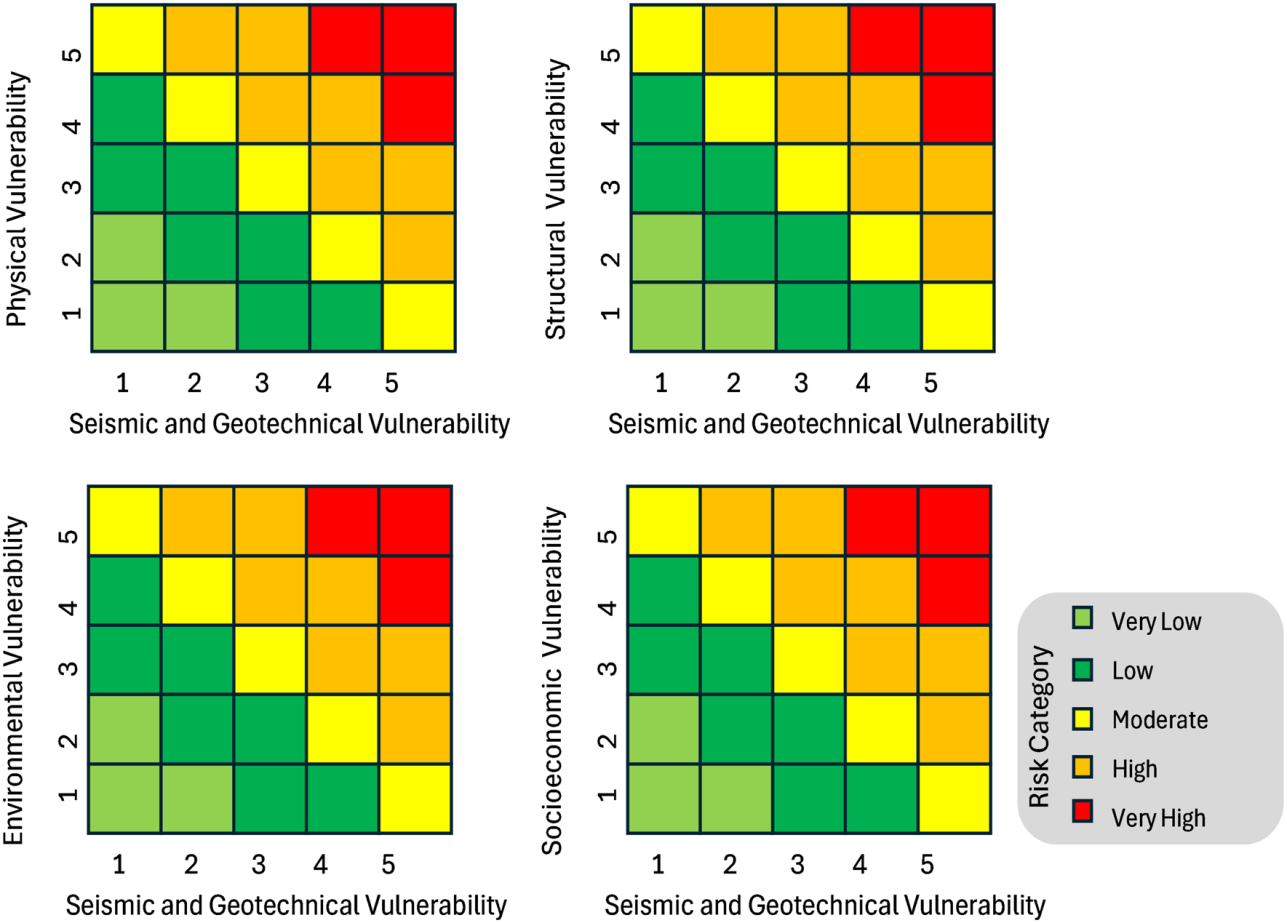
Integrated seismic risk matrices incorporating the identified vulnerabilities in the present study.

Considering seismic and geotechnical vulnerability together is essential for a comprehensive understanding of risk, as it reveals how ground conditions and earthquake hazards interact with and amplify various types of vulnerabilities. The risk matrices presented in Fig. 20 highlight a complex interplay between seismic and geotechnical vulnerability and various selected secondary level vulnerabilities that influence overall disaster risk. Additionally, environmental vulnerabilities can further increase exposure to hazards^71^. Integrating seismic and geotechnical data allows for risk assessment, guiding targeted interventions like improved building codes, land-use planning, and disaster preparedness strategies that address multiple layers of vulnerability simultaneously.

**Fig. 21:**
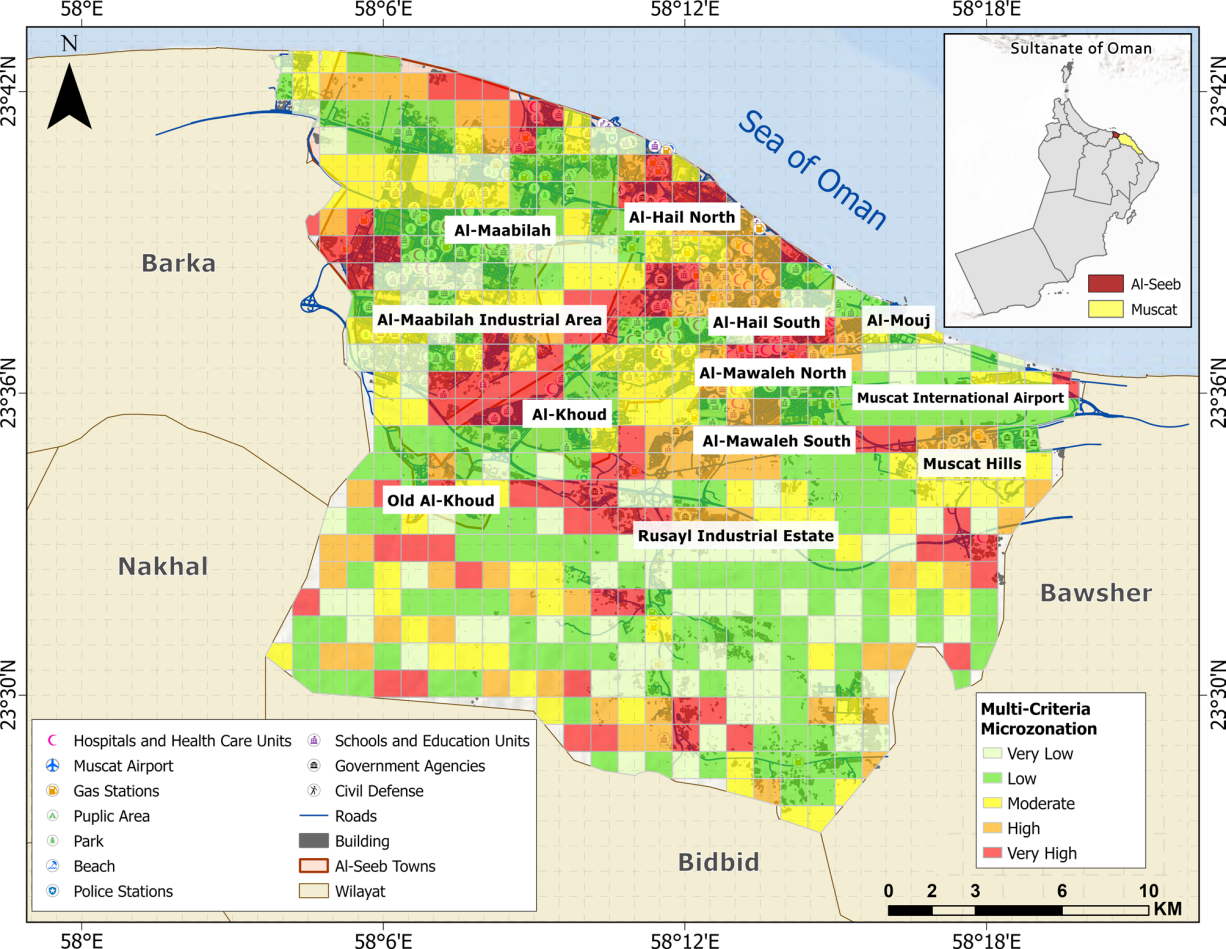
Microzonation map illustrating seismic risk zones in Al-Seeb. The base map is quoted from the publicly available Google satellite imagery maps (https://maps.google.com/).

Using the risk matrices shown in Fig. 20 as a reference, a risk index was computed for each grid, dividing the Al-Seeb region into five distinct risk zones, which are depicted in the map presented in Fig. 21. The central urban zones such as Al-Hail North and parts of Old Al-Khoud are marked in red, indicating very high vulnerability.

In contrast, some parts of Al-Mawaleh North showed a mix of orange and red zones, posing considerable danger due to comprise of structural aspects in terms of limited retrofitting of existing structures. Moving toward the parts of Muscat Hills displayed predominantly yellow zones, reflecting moderate vulnerability. These regions benefited from more stable subsurface conditions, which lower the likelihood of severe ground failure. Despite this, Rusayl industrial estate introduced specific risks related to hazardous facilities and critical infrastructure that could be impacted even by moderate shaking. Overall, this map highlights the need for location-specific mitigation strategies, with high-risk urban cores requiring urgent upgrades to land-use policies, while peripheral zones should focus on maintaining their relative stability through sustainable development and earthquake hazard awareness planning.

**Fig. 22:**
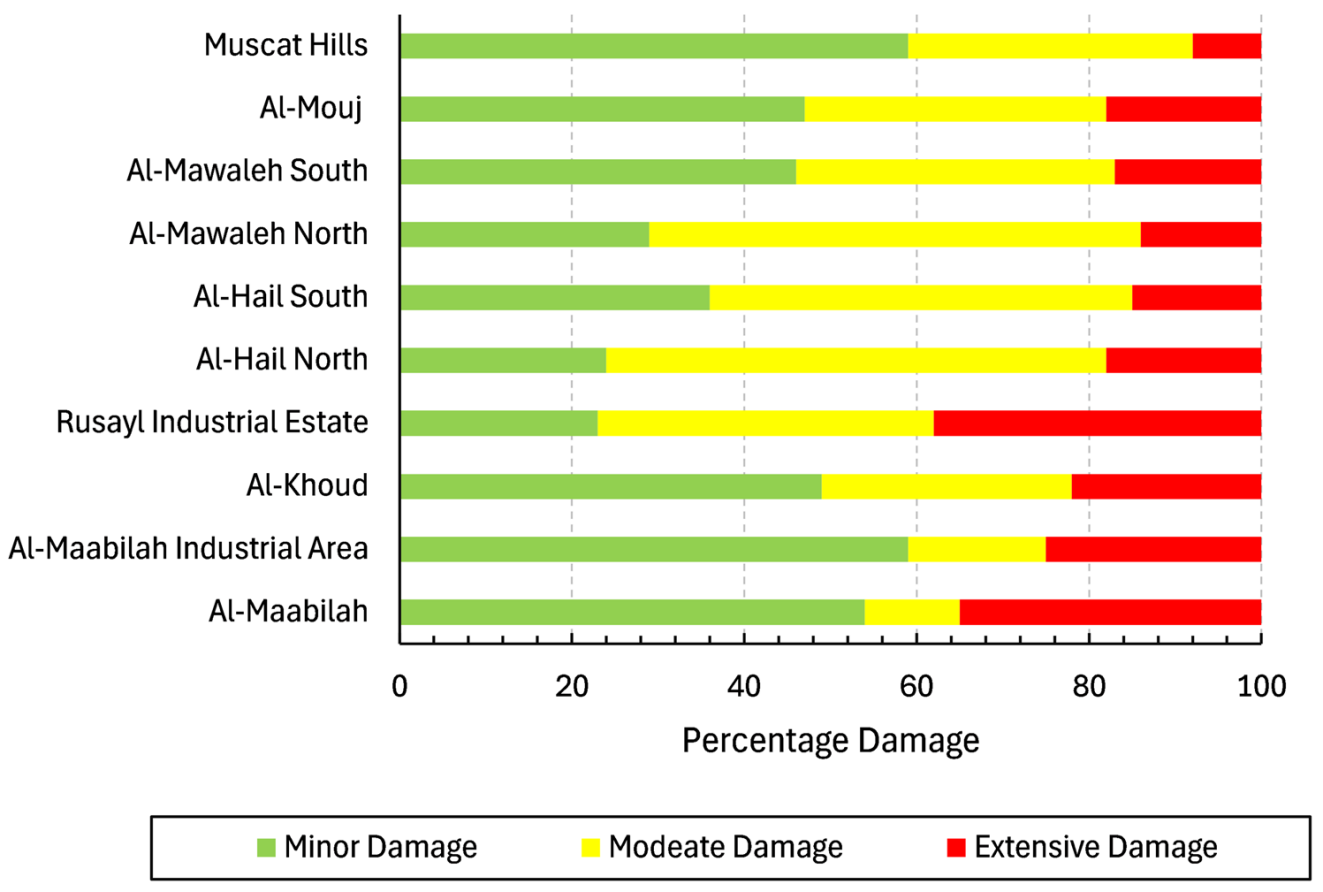
Damage assessment for selected localities in Al-Seeb.

Figure 22 presents a comparative analysis of the percentage damage to buildings across various areas. Muscat Hills has the lowest building damage, with 60% experiencing minor damage and only 10% extensive, indicating strong resilience due to modern construction. In contrast, Rusayl industrial estate is the most vulnerable, with 40% of buildings facing extensive damage, likely due to aging infrastructure and industrial characteristics. Building units in areas like Al-Mawaleh, Al-Hail, and Al-Khoud showed moderate vulnerability, with 50 to 60% of buildings sustaining moderate damage, reflecting mixed construction quality. With 60% experiencing only minor damage and just 20% sustaining extensive damage, the performance suggested a certain degree of structural resilience in Al-Maabilah buildings. These comparisons highlighted the need for targeted retrofitting in high-risk areas to reduce seismic vulnerability.

Compared to previous seismic microzonation studies conducted in metropolitan areas such as Istanbul^72,73^, Kathmandu^74^, and Tehran^75–77^, the vulnerability assessment of Al-Seeb aligns with common findings where structural deficiencies and geotechnical conditions are primary determinants of seismic risk. However, Al-Seeb’s vulnerability profile uniquely reflects a higher relative influence of proximity to dangerous industrial facilities, a factor less emphasized in many traditional urban microzonation^78–81^ studies but critical due to the region’s specific land use pattern. Similar to these global studies, the integration of multi-hazard themes through GIS-AHP allows for a comprehensive spatial representation of vulnerability patterns, facilitating targeted mitigation strategies.

Nevertheless, inherent uncertainties must be acknowledged. The subjectivity involved in expert judgment for criteria weighting within AHP introduces potential bias, which could affect the robustness of theme prioritization. Additionally, reliance on RVS survey data and secondary sources may lead to classification errors or omissions, particularly in fast-developing urban zones. Therefore, results should be interpreted with caution, and periodic data updates combined with ground-truthing are recommended to enhance reliability and support dynamic urban planning decisions.

## Conclusions

This study introduces a comprehensive and innovative framework that integrates the AHP with GIS-based spatial analysis to assess seismic vulnerability in the Al-Seeb region of Muscat. MCDM approach is used employing RVS survey to capture critical structural parameters across residential zones. This methodological innovation directly supports Oman Vision 2040’s goals for modern, evidence-based urban planning and the development of resilient, data-driven infrastructure systems, in line with UN-SDG 11 (sustainable urbanization). Al-Mawaleh North and South, along with Al-Hail North and South, consistently demonstrated high physical vulnerability. These patterns suggested deficiencies in transport connectivity and access to vital facilities, raising concerns related to emergency healthcare and disaster preparedness under post-disaster recovery conditions (MH and DRU indicators). By mapping risk zones, the study informs smarter land-use policies, which promotes integrated policies for disaster risk management in urban planning.

Table 6 enlists area-specific policy recommendations for retrofitting, infrastructure investment, and emergency planning across Al-Seeb, aligning with UN-SDG 11 targets and Oman Vision 2040 objectives for sustainable urban development. The present study framework establishes a scalable and transferable methodology for seismic risk governance in rapidly urbanizing regions, particularly in data-sparse environments. This work supports the sustainable society agenda by providing a risk-informed framework that can guide resilient urban planning in emerging cities like Sultan Haitham City and Yiti City. By harmonizing spatial analytics with policy-driven vulnerability themes, it sets a precedent for global application in enhancing resilience planning across coastal cities, bridging local risk priorities with international sustainable development and climate adaptation agendas.

**Table 6:** Summary of localized policy recommendations for selected areas in Al-Seeb region.

Area	Retrofitting focus	Infrastructure investment	Emergency planning priority
Al-Hail North	Strengthen old housing blocks; prioritize non-engineered structures	Develop road connectivity and storm drainage near coastal blocks	Community-based training and multi-hazard drills (UN-SDG 11.5)
Old Al-Khoud	Upgrade aging residential units; seismic retrofitting of heritage sites (UN-SDG 11.4)	Upgrade lifeline services (water, electricity)	School evacuation drills and civil defense coordination (UN-SDG 11.b)
Al-Mawaleh North	Moderate retrofitting of mixed construction types	Expand hospital access and strengthen feeder road networks	Map emergency routes and install early-warning systems (UN-SDG 11.5)
Al-Khoud	Reinforce public buildings and schools	Improve transport and utility corridors under rapid expansion	Public awareness programs and shelter zoning
Al-Maabilah	Target informal settlements for seismic resilience	Increase flood drainage and sanitation infrastructure in low-lying areas	Hazard signage and training for informal sectors
Al-Maabilah industrial area	Industrial warehouses and utility structures need seismic compliance upgrades	Enhance fire-fighting infrastructure and road access in industrial zones	Industrial safety audits and chemical spill preparedness (UN-SDG 11.6)
Al-Mawaleh South	Strengthen community buildings and clinics	Improve stormwater outlets and health infrastructure	Health emergency planning and vulnerable group outreach
Al-Hail South	Reinforce residential blocks with poor structural grading	Expand resilient utility grids and emergency water supply	Integrate neighborhood emergency maps and apps (UN-SDG 11.3)
Muscat Hills	Low priority; monitor for hillside slope stability	Maintain low-density development with smart infrastructure	Smart hazard monitoring with automated alerts (UN-SDG 11.3, 11.c)
Rusayl Industrial estate	Mandatory structural audits of industrial units and energy facilities	Modernize logistics corridors, fuel lines, and emergency exits	Industrial hazard simulations and first-responder drills (UN-SDG 11.b)

The risk assessment presented in this study, while comprehensive, involves several limitations and potential uncertainties across different vulnerability dimensions. Seismic vulnerability estimations rely on available ground motion parameters, which may not fully capture local amplification effects or near-surface variability due to limited site-specific recordings. Geotechnical vulnerability is influenced by interpolated soil data, which may vary considerably in microzones, especially in reclaimed or poorly mapped areas. Physical vulnerability assessments, including building age and typology, are subject to generalization due to incomplete structural records and limited post-construction evaluations. Structural vulnerability indices, particularly for older or undocumented buildings, carry uncertainty due to assumptions about material strength and design standards. Environmental and physical vulnerability, such as risks from secondary hazards may be underestimated due to the lack of integrated hazard interaction modeling. Socioeconomic vulnerability reflects census-based data, which may not accurately represent transient populations, informal settlements, or recent urban developments. These factors together underscore the need for continued field validation, refined data integration, and probabilistic modeling to reduce uncertainty in future assessments.

A similar type of work can also be conducted for other rapidly developing cities, such as Duqm, Salalah, and Sohar. These regions are witnessing major government-led infrastructure initiatives, for example, the Duqm Special Economic Zone (SEZAD), the Salalah Free Zone and port expansion, and the Sohar Industrial Port and Freezone development. Integrating risk and vulnerability assessments into such strategic projects would significantly enhance their long-term resilience and sustainability. Future work can incorporate probabilistic. seismic hazard analysis (PSHA) to capture the uncertainty of seismic inputs more rigorously, enabling scenario-based vulnerability modeling. Additionally, machine learning algorithms can be employed to refine and validate the spatial distribution of vulnerability by learning from historical damage patterns, remote sensing data, and structural typologies, thereby enhancing the predictive accuracy of the current framework.

## Data Availability

The datasets generated during and/or analyzed during the current study are available from the corresponding author on reasonable request.
